# A PDK-1 allosteric agonist neutralizes insulin signaling derangements and beta-amyloid toxicity in neuronal cells and in vitro

**DOI:** 10.1371/journal.pone.0261696

**Published:** 2022-01-21

**Authors:** Henry Querfurth, John Marshall, Keykavous Parang, Mengia S. Rioult-Pedotti, Rakesh Tiwari, Bumsup Kwon, Steve Reisinger, Han-Kyu Lee

**Affiliations:** 1 Department of Neurology, Tufts Medical Center, Boston, MA, United States of America; 2 Department of Molecular Pharmacology, Physiology, and Biotechnology, Brown University, Providence, RI, United States of America; 3 Center for Targeted Drug Delivery, Chapman University, School of Pharmacology, Irvine, CA United States of America; 4 Department of Neurology, Clinical Neurorehabilitation, University of Zurich, Zurich, Switzerland; 5 Department of Neurology, Rhode Island Hospital, Providence, RI, United States of America; 6 Med Chem Partners, Lexington, MA, United States of America; H Lee Moffitt Cancer Center and Research Institute, UNITED STATES

## Abstract

The Alzheimer’s brain is affected by multiple pathophysiological processes, which include a unique, organ-specific form of insulin resistance that begins early in its course. An additional complexity arises from the four-fold risk of Alzheimer’s Disease (AD) in type 2 diabetics, however there is no definitive proof of causation. Several strategies to improve brain insulin signaling have been proposed and some have been clinically tested. We report findings on a small allosteric molecule that reverses several indices of insulin insensitivity in both cell culture and *in vitro* models of AD that emphasize the intracellular accumulation of β-amyloid (Aβi). PS48, a chlorophenyl pentenoic acid, is an allosteric activator of PDK-1, which is an Akt-kinase in the insulin/PI3K pathway. PS48 was active at 10 nM to 1 μM in restoring normal insulin-dependent Akt activation and in mitigating Aβi peptide toxicity. Synaptic plasticity (LTP) in prefrontal cortical slices from normal rat exposed to Aβ oligomers also benefited from PS48. During these experiments, neither overstimulation of PI3K/Akt signaling nor toxic effects on cells was observed. Another neurotoxicity model producing insulin insensitivity, utilizing palmitic acid, also responded to PS48 treatment, thus validating the target and indicating that its therapeutic potential may extend outside of β-amyloid reliance. The described *in vitro* and *cell based-in vitro* coupled enzymatic assay systems proved suitable platforms to screen a preliminary library of new analogs.

## Introduction

Clinically-based Alzheimer’s Disease (AD) currently affects 5.8 million or 1 in 10 adults (10%) in the U.S.A. over age 65 and 32% in the >85 age group. Several phase III clinical trials of promising agents to prevent AD progression, based primarily on the amyloid hypothesis, have yielded disappointing overall results. These included anti-amyloid agents such as γ-secretase (Semagacestat) [1] and BACE (Verubecestat) [2] inhibitors and passive immunotherapies (Bapineuzumab, Gantenerumab, Solanezumab, IVIG) [3–6]. However, some recent positive outcome measures resulting in an FDA approval, with allowance for study design issues (Aducanumab), support continued efforts (e.g. Donanemab) [7, 8]. These drug trial outcomes call for targets that are not only based on the supply side or removal of secreted β-amyloid (Aβ), but directly address its toxic effects on critical neuronal metabolic, plasticity and survival signal pathways. One less often considered offender, intra-neuronal β-amyloid peptide (Aβi) accumulation, may be relevant to the early pathogenesis of AD [9–11] and considered a target. A related and potentially remediable inciting risk factor for both amyloid formation and AD progression is systemic insulin resistance (IR) and type 2 diabetes mellitus (T2DM) [12]. Moreover, there is wide recognition that the AD brain is itself an insulin resistant end-organ, a so-called ‘type III diabetes’ condition [13, 14].

While the relationship of brain insulin resistance to the accumulation of cellular and plaque Aβ and the cross-talk between T2DM, peripheral and central IR continue to be explored, there is ample experimental data that peripheral IR can drive Alzheimer pathology. Animal models of T2DM or bearing an AD transgene and made pre-diabetic on an oil rich or high fat diet, show ensuing brain IR and amyloidogenesis [15–17]. Loss of peripheral insulin signaling can further result in the central hyperphosphorylation of Tau [18, 19]. Targeted disruption of insulin signaling within the CNS by genetic means or intracerebral streptozotocin injection also leads to AD-like degeneration and tau hyperphosphorylation [20–22]. In turn, these pathologic changes are rescued by insulin treatments.

The essential roles of brain insulin and the mechanism behind IR in AD have been extensively studied (for reviews [23, 24]). Levels of insulin, insulin-like growth factor I (IGF-I)and cognate receptors, become deregulated in AD brain [25–29]. Normally, these promote energy metabolism, neuronal survival, synaptic plasticity [30] and memory formation [31, 32]. Insulin/IGF-1 receptors populate synapses [33] where they signal through IRS-1 (insulin receptor substrate-1) and the phosphatidylinositol 3-kinase / Protein kinase B (PI3K/Akt) and MAPK pathways [34]. The insulin-PI3K/Akt activation sequence brings together phosphoinositide-dependent protein kinase-1 (PDK-1) and Akt in a sub-membrane complex [35]. Activated Akt maintains post-mitotic cell viability by phosphorylating several pro-apoptotic mediators, including glycogen synthase kinase-3β (GSK-3β) [36, 37]. Conversely, dephosphorylation (inhibition) of Akt sensitizes the cell to environmental stressors [38, 39]. IGF-1/Akt regulate transcription factors to support hippocampal progenitor neurogenesis [40, 41] and learning/memory, e.g. via CREB [42, 43]. Insufficient insulin signaling impacts the activity of mTOR, a suppressor of autophagy, lowers levels of IDE, known to degrade Aβ [17, 44] and negatively affects translocation of GLUT-3/4, glucose transporter proteins [45, 46].

Aβ42 accumulation in excess produces neuronal IR and PI3K/Akt axis disruption by several mechanisms. The inhibitory effect of Aβ42 oligomers on hippocampal LTP and PI3K/Akt is reversed by insulin [47, 48]. One mechanism is the caspase-mediated cleavage of Akt1 [49]. Next, extracellular Aβ inhibits the binding of insulin to its receptor [50] and results in their downregulation (removal) from the neuronal membrane [47, 51]. Aβ oligomers are further shown to inhibit insulin-induced phosphorylations of both insulin receptor [51] and Akt [48]. Lastly, IR in AD brain is linked to inhibitory feedback phosphorylations of IRS-1 (S616 and S636) by pS6K [27, 52]. Aβ provokes IR in this way by first activating (de-repressing) mTOR via phosphorylation of the inhibitory subunit, PRAS40 [53]. mTOR target pS6K becomes indirectly stimulated [53–55]. The end result is a decrease in IRS-1 levels [27, 56]. Interestingly, the inflammatory cytokine TNFα mediates the same outcome [57, 58].

PI3K/PDK/Akt signaling in AD brain is reported to be abnormally stimulated [52, 59–63]. Accordingly, over-activation of downstream mTORC1 is found [28, 64–67] alongside loss of autophagy markers [68, 69]. Intra-hippocampal injections of anti-Aβ antibody or immunization normalized the hyper-activation of Akt and mTOR in transgenic AD mice [53, 70]. The mechanisms underlying the paradoxical hyperactivity of Akt and mTOR under basal conditions, are not completely understood. In addition to direct activation of mTOR noted above, Aβ can directly inactivate PTEN (phosphatase and tensin homolog), thereby disinhibiting PI3K [69, 71]. One seminal study found all IRS-1 -S and -Y sites were hyperphosphorylated in live AD hippocampal and cerebellar tissue, essentially isolating IRS-1 from binding to insulin receptors and p85-PI3K. Intrinsic over-activation of mTOR/S6K and other kinases was held responsible. Importantly, their work proved resistance to insulin/IGF-1 action in AD; a 90% decrease in Akt, IRS-1, IR, and mTOR phospho-activations in response to insulin stimulation [52].

There is opposing evidence gathered from several AD models that basal Akt is *deactivated*, which is also consistent with IR in AD. Inhibited Akt is further noted in post-mortem tissue from AD [64, 72], Huntington’s and Parkinson’s diseases [67, 73–75]. In two AD models, the inhibition of PTEN instead rescued synaptic and cognitive impairments, mediated through the stimulation of PI3K/Akt [76]. Conversely, PTEN over-expression led to synaptic depression. Aβ peptides applied to hippocampal neurons induced the same synaptic defects and dephosphorylation of Akt by recruiting PTEN to dendritic spines [76]. In 2576 AD mice, where cellular Aβ is co-localized to mTOR, it was actually found to have an inhibitory role [72]. Moreover, reduction in mTOR signaling markers and basal phospho-Akt levels/enzymatic activities, were found in 2xAPP/PS1 mice and in AD brain. These were correlated with oxidatively damaged synaptic Akt. Akt enhancement rescued BDNF-induced protein translation [77]. Deactivation of Akt is reported in rat PCNs and N2a cells exposed to oligomeric Aβ, resulting in inhibition of normal BDNF-induced Akt/mTOR activation [78, 79].

Due to conflicting reports and the paucity of preclinical and clinical data on direct Akt/PDK-1 intervention in AD models, we tested the hypothesis that targeting insulin resistance at this step may be beneficial. In a previous study, intraneuronal Aβ42 (Aβi) expression led to a decrease in the levels of p-Akt and activity, causing p-Tau accumulation and apoptosis [80]. Aβi inhibited the association of PDK-1 with Akt, resulting in the loss of normal insulin-stimulated pathway activation [64, 81]. This added mechanism for IR presents a novel target for the treatment of AD. We reasoned that an allosteric ligand acting on the Akt/PDK-1/mTORC2 interaction complex could normalize insulin sensitivity and restore the imbalance in Akt activity. Promising results from early clinical trials in MCI and mild AD of insulin sensitizers (metformin, [82]), GLP-1 receptor agonist/ incretin analogs (liraglutide, [83]), intranasal (IN) insulin [84, 85] and insulin- sensitizing PPAR-γ agonists that target genes such as IRS-1, GLUT-4 and PI3K [86, 87] (Rosiglitazone, [88]; Pioglitazone, [89]), support finding druggable targets in this pathway and several relevant ongoing trials: Metformin (phase 3, NCT04098666), liraglutide (phase2b, NCT 01843075, [90]) and semaglutide (phase 3, NCT 04777396). However, some phase 3 trials have not met their primary endpoints or been terminated for lack of efficacy, e.g. IN insulin [91], Rosiglitazone [92, 93] and Pioglitazone [94, 95]. Acknowledging this uncertainty, an approach to reestablish insulin sensitivity in AD need not be dependent on the amyloid hypothesis or any of these interventions in particular to be useful. We report preclinical findings using PS48, a chlorophenyl pentenoic acid and allosteric activator of PDK-1 [96, 97].

## Materials and methods

### Ethics

Animal research ethics for this work were approved by the IACUC at Rhode Island Hospital under Clinical, Biochemical and Electrophysiologic Investigations into Neurodegeneration, no. 462439-3/2013, PI: HWQ. All personnel and collaborators involved in these experiments, performed on mice and rats, were included. All procedures, tissue collection, biohazard uses, recombinant DNA, husbandry, special diets, breeding and euthanasia were covered. Euthanasia was by either sodium pentothal (120–200 mg/Kg IP) or ketamine (80–100 mg/Kg IP) followed by decapitation.

### Cell culture and reagents

Undifferentiated neuroblastoma cell lines SH-SY5Y (human) and N2a (mouse) (ATCC, Manassas, VA; Sigma, St. Louis, MO) were grown in DMEM, 10% FBS, 25mM glucose, at or below 80% confluence. SH-SY5Y were left undifferentiated. Mouse C_2_C_12_ cells (ATCC) were grown in Dulbecco’s modified Eagle medium (DMEM), 20% fetal bovine serum (FBS) (Invitrogen), and maintained for passage below 60% confluence. Cultures at or above 90% confluence were then differentiated in DMEM, 2% adult horse serum (DM) for 3 days before use. Primary rat cortical neurons (PCNs) were cultured from E18 Sprague-Dawley rat fetal cortex (Charles River, Wilmington, MA) as described [98]. Briefly, isolated fetal cerebral cortex was dissociated into single cells and then seeded into 6-well plates coated with poly-D-lysine at 1 × 10 ^6^ cells per well. PCNs were cultured in neurobasal medium (Invitrogen, Carlsbad, CA) containing 2% B27 without insulin, 25 mM D-glucose, 0.5 mM L-glutamine and 1% penicillin/streptomycin for 7 days before experiments.

Antibodies used were: goat anti-Akt-1 and Actin (Santa Cruz Biotechnology); anti-p-Akt (Ser473 and Thr308), p-GSK-3α/β (Ser21/9), and GSK-3α/β (Cell Signaling); mouse anti-PDK-1 (BD Biosciences); 6E10 (Covance, Co); R1282 (gift from Dr. D. Selkoe); anti phospho-CREB (Ser 133) 87G3 rabbit mAb Cell Signaling #9198); anti-mTOR (Cell Signaling, recognizing C1 and C2), p-mTOR (Ser2448, Ser2481), neuron-specific enolase (NSE, SantaCruz), 6E10 (anti Aβ, Covance). GSK-3 fusion peptide (crosstide-paramyosin), a 27 kDa substrate for Akt phosphorylation (Cell Signaling #9237). See S1 Table for dilutions. Fluorophore-labeled Aβ42 is carboxy-fluorescein conjugated to the N’ terminus (FAM-Aβ42) purchased from Anaspec (Fremont CA). Recombinant human Akt1 (inactive) and PDK-1 (active) proteins were obtained from Amsbio. PS48 and PS47, from SIGMA and Axon MedChem; dipalmitoyl-PIP3 (Matreya, State College, PA); ATP (Adenosine-5’-triphosphate, disodium salt) is supplied as a 10 mM solution in doubly distilled water (Cell Signaling). Protein A/G PLUS-Agarose (Roche). Human Insulin, recombinant, dry or 10 mg/ml solution, was purchased from Sigma-Aldrich.

### Infection of SY5Y and C_2_C_12_ myotubes with adenoviruses

Adv TetOn and TRE-Aβ42 viruses were described previously [80]. SY5Y and C_2_C_12_ myotubes were infected with Adv Aβ42/TetOn (4:1 ratio) 24~36 hr before doxycycline induction (1 μg/ml) for an additional 24~36 hr. Insulin (10 or 40 nM) was added in the last 20–30 min before harvest. PS48 (Sigma), PS47 (Axon Medchem, Reston VA), and 501-1-x compounds were added 5 min before adding insulin. Cell extracts were prepared in lysis buffer [20 mM Tris-HCl (pH 7.5), 150 mM NaCl, 1 mM EDTA, 1 mM Na3VO4, 1% NP-40, 10% Glycerol, 1 mM Na4P2O7, 1 μg/ml Leupeptin, 1 μg/ml Pepstatin A, 1 μg/ml Aprotinin, 0.1 mM phenylmethylsulfonyl fluoride (PMSF), and protease inhibitor cocktail (Roche)] and were stored at -80°C until use.

### Cell viability

SH-SY5Y Cells were washed twice in warm DPBS and incubated in 1 ml DMEM containing 0.5 mg (3-[4,5-dimethylthiazol-2-yl]-2,5-diphenyltetrazolium bromide) (MTT or WST; Molecular Probes, Eugene, OR) for 2–3 h at 37°C and 5% CO2. The medium was aspirated and the cells were washed twice with pre-warmed DPBS. The formazan salts were dissolved in 1 ml pure ethanol before use. Cells were homogenized by repetitive pipetting and centrifuged for 5 min at 4500 rpm, and the supernatant collected. Absorbance was read against an ethanol blank at 590 nm.

### Aβ and ADDL preparation

Aβ peptides were obtained from BioSource as dried trifluoroacetic acid salts. Monomeric Aβ peptides were prepared by solubilization in 5% dimethyl sulfoxide (DMSO); 25 mM Tris-HCl, pH 7.4, and used fresh or flash frozen. Aβ-derived diffusible ligands (ADDLs) were prepared as detailed previously [64] and as according to Lambert et al., [99] and Klein et al., [100]. Briefly, Aβ peptide was dissolved in 1,1,1,3,3,3-hexafluoro-2-propanol (Sigma) and evaporated on a Speedvac. The Aβ film was resuspended in 100% anhydrous DMSO, diluted to 5mM in F12 medium lacking phenol red (BioSource), and incubated at 4°C for 24 to 48hr. Following incubation and centrifugation at 14,000 g for 10 min at 4°C, the supernatant containing ADDL-enriched Aβ was transferred to a new tube.

### Western blot analysis

Whole-cell extracts were used directly for western blot analysis (20~30 μg). Extracts from cultured cells prepared in lysis buffer, were diluted into Laemmli sample buffer, heated (95°C, 10 min), cleared by centrifugation, separated on SDS–PAGE and transferred to PVDF membrane (Immobilon-P; Millipore). Membranes were blocked in TBS containing 0.3% Tween-20 and 5% (wt/vol) non-fat dry milk. After incubation with primary antibodies (18 hr at 4°C in buffer containing 5% BSA and 0.05% NaN3), blots were washed and incubated in HRP-conjugated secondary antibodies (1:2000 dilution; Cell Signaling). Signals were detected using ECL reagents and quantified using a Kodak Image Station 4000R.

### In vitro p-Akt and activity levels

Immunoprecipitations (IPs) of PDK and Akt1 were prepared from 100 μg of either SH-SY5Y, C_2_C_12_ myotubes or from insulin-treated cultures. Alternatively, commercial recombinant Akt (100 ng) and PDK (10ng) proteins were used. PIP3 (50 nM) in the role of activating phosphoinositide lipid, GSK-3β-paramyosin fusion protein (1 μg/50 μl, 1.0 μg), kinase buffer and synthetic Aβ42 peptide oligomers were added. ATP (200 μM) started the reaction (50 μl) that continued for 30 minutes at 30°C. The reaction was stopped by adding 40 μl of Laemmli buffer. 20 μl of sample was loaded onto a 10% polyacrylamide gel.

An *in vitro* radio assay (EMD Millipore, KinaseProfiler) was also adapted as follows. PKBα (human, recombinant, inactive, 209 nM) is incubated in 8 mM MOPS pH 7.0, 0.2 mM EDTA with 30 μM GSK3α/β consensus sequence GRPRTSSFAEGKK and PDK1 (human, recombinant, 285 nM). β-amyloid peptide (oligomerized, 5 μM final) and PS48 are added. Final DMSO is 2%. 10 mM Mg Acetate and [γ-33P- ATP] (specific activity approx. 500 cpm/pmol) are prepared. The reaction is initiated by the addition of the Mg ATP mix (200 μM ATP final). After incubation for 40 minutes at 37°C, the reaction is stopped by adding 3% phosphoric acid solution. 10 μl of the reaction is spotted onto a P30 filtermat and washed three times for 5 minutes in 75 mM phosphoric acid and once in methanol, prior to drying and scintillation counting.

### Electrophysiology

Minor modifications were made to a previously published procedure [101, 102]. Deeply anesthetized rats (pentobarbital, 50 mg/kg) were decapitated, their brains quickly removed and immersed in cold (5–7°C), oxygenated (95% O2/5% CO2) artificial cerebrospinal fluid (ACSF) containing (in mM): 126 NaCl, 3 KCl, 1.25 NaH2PO4, 1 MgSO4, 2 CaCl2, 26 NaHCO3, 10 dextrose. Coronal slices of prefrontal cortex from day 14 rat pups (400 μm) were perfused with Aβ oligomers (40 nM) or Aβ plus PS48 (10 μM) x 60 min. before applying the high frequency stimulation (HFS) protocol. Control treatment is DMSO in ACSF (artificial cerebrospinal fluid, 20 μM bicuculline). Extracellular postsynaptic field potentials were recorded using an AxoClamp2B amplifier (Axon instruments) and EX1 differential amplifier (Dagan), and digitized at 10 kHz. Data was acquired using Igor Pro (Wave Metrics) and Neuromatic (www.neuromatic.thinkrandom.com). The stimulus intensity eliciting 50% of the maximum amplitude (~32 μA) was used for all measurements before and after LTP induction. Baseline amplitudes were recorded for 20–30 minutes using single field stimuli applied every 30 sec (2Hz) to layer lV-V using concentric bipolar electrodes. Following a stable baseline period, LTP was induced by two sets of high-frequency stimulation (HFS) at 100 Hz, 60 μA (twice stimulus intensity), for 1 sec, 20 sec apart. Extracellular postsynaptic field potentials were measured from layer ll-lll using glass micropipettes filled with 0.9% NaCl. The amplitude rather than the slope of evoked FPs was used as a measure of the population excitatory synaptic response because in the neocortex the initial slope is contaminated by antidromic stimulation. The last 10 minutes (20 points) of the stimulated EPSP recordings were normalized to baseline and then averaged. LTP values were expressed as a percentage of mean baseline EPSP ± SEM or % change normalized to baseline. ANOVA with Tukey’s multiple comparisons and paired two-tailed t-tests were used for statistical analysis.

### Binding in solution: Fluorescence polarization

A procedure based on Lynch et al., 1997 [103] was modified and adopted from Tiwari et al. [104]. FAM-labeled Aβ42 peptide (probe) is mixed with wild type Aβ42 (1 mM total in HFIP) in a 1:2 molar ratio, evaporated to film, then solubilized to 5 mM in 100% DMSO and bath sonicated. It is then diluted to a 100 μM stock in HAMS F12, pH7.4, 2% DMSO and incubated 4° C for 24 hrs to oligomerize Aβ (checked by western blot against 6E10 and directly by UV light-western). The recombinant protein binding targets, PDK-1 (59 kDa) or Akt-1 (60 kDa) are stocked as 500 μM. Final probe concentration is fixed at 200 nM. Final target concentrations (0–10 μM) are increased in successive samples until saturation is reached. The reaction is carried out manually in 0.6 cc quartz cuvettes (Suprasil Micro cells, 5 mm path length) for 30 min, 25°C, in 1X buffer: 100 mM NaCl, 20 mM phosphate pH7.4, 2 mM DTT, 0.1% BSA, 2% DMSO. Final volume 600 μl. Where PS48 was added, final concentrations tested were 10, 20 and 100 μM. Absolute fluorescence polarization (FP) values were read off a LS55 PerkinElmer luminescence spectrophotometer, excitation 485/ emission 530 nm. FAM-Aβ42 was prepared as ADDLs and incubated with recombinant Akt-1 or PDK-1 (final [Aβ42] 200 nM, Akt from 0 to 12.5 μM. Immunoprecipitation of Aβ42 with 6E10 and western developed with anti Akt and re-probed with R1282.

### Drug screening

Two focused libraries of PS48-family compounds were designed based on known and hypothesized structure-activity relationships and tested using an *in vitro* screen carried out in 48 well plates as follows (in order of rapid additions, final concentrations): 10x Kinase buffer, recombinant PDK-1 (5 μl, pre-immunoprecipitated onto agarose beads using monoclonal IgG), PIP3 50 nM, Aβ42 (as ADDL oligomers, 10μM), recombinant Akt-1 (5 μl, pre-immunoprecipitated onto beads using polyclonal goat IgG, treated with PP2A to dephosphorylate Akt and washed), compound or PS48/ PS47 (solubilized in DMSO then diluted with H2O, 10 μM), ATP (to initiate Akt activation, 200 μM). Incubation proceeded for 15 min. GSK-tide (Cell Signaling) was then added and the reaction allowed to carry for 20 min more before termination in sample buffer and fractionation on SDS gel. Transfers were probed with anti-p-GSK, anti phospho-473 and -308Akt and total Akt. The *in vitro* results were validated using a cell culture-based assay, as above. Briefly, adenovirus-infected PCNs (2 days) were induced with doxycycline (48 hrs) to express Aβ42. The compound was added for 12 hrs. The cells were stimulated with insulin before harvest.

### Statistical

Where quantified, experiments were carried out in triplicate unless otherwise stated. Mean, standard errors and significance levels using students t- test were computed in Excel or Prism. *In vitro* Akt activation assay data (see above), in which the % inhibitory effects of Aβ42 monomers and ADDLs were tested, was fitted using a 2 site (hyperbolic), non-linear algorithm (Prism) to obtain Imax and K0.5 equilibrium constants. Western signal intensities were all quantified by densitometry. Akt activation (phosphorylation) and activity (GSK phosphorylation) western results (stimulation or inhibition) were for the most part, concordant and equivalent in the fraction of change versus control. Therefore, where both endpoints were evaluated, their normalized values were combined in the quantification as indicated. ANOVA (1 way; between treatment groups or columns and 2 way; between treatments groups and between repeated measures or rows) was carried out on Western, cell viability and LTP experiments. Where indicated, Dunnett’s or Tukey’s multiple comparisons were applied. Effect sizes for Aβ and drug additions are given as mean differences (± SE difference) and 95% confidence intervals.

## Results

We had previously shown that cellular β-amyloid expression inhibits PI3K-PDK1-Akt signaling [80, 81, 105]. To summarize, *in vivo* assays of phospho-Akt/total Akt and downstream substrate, phospho-GSK3β levels were carried out on extracts from cultured neurons exposed to an inducible adenoviral vector encoding Aβ42 [64, 80]. Cells were pretreated with insulin for 20 min prior to harvest in order to activate Akt, finding that insulin-stimulated p-Akt levels were reduced to baseline in the presence of Aβ42 expression. To confirm this, Akt enzymatic activity was measured in cell lysates using a coupled assay; immuno-precipitated (IP) Akt from insulin-stimulated cells, phosphorylated a synthetic substrate peptide bearing the phosphorylation consensus sequence GSK3β fused to paramyosin (crosstide). An *in vitro* kinase assay was also developed (see methods), finding that 5 μM Aβ peptide inhibited the Akt-dependent phosphorylation of the target substrate, accompanied by an expected reduction in pSer473Akt. Further data [64] indicated that Aβ inhibits the PDK-1- dependent activation of Akt by disrupting their interaction.

**Fig 1:**
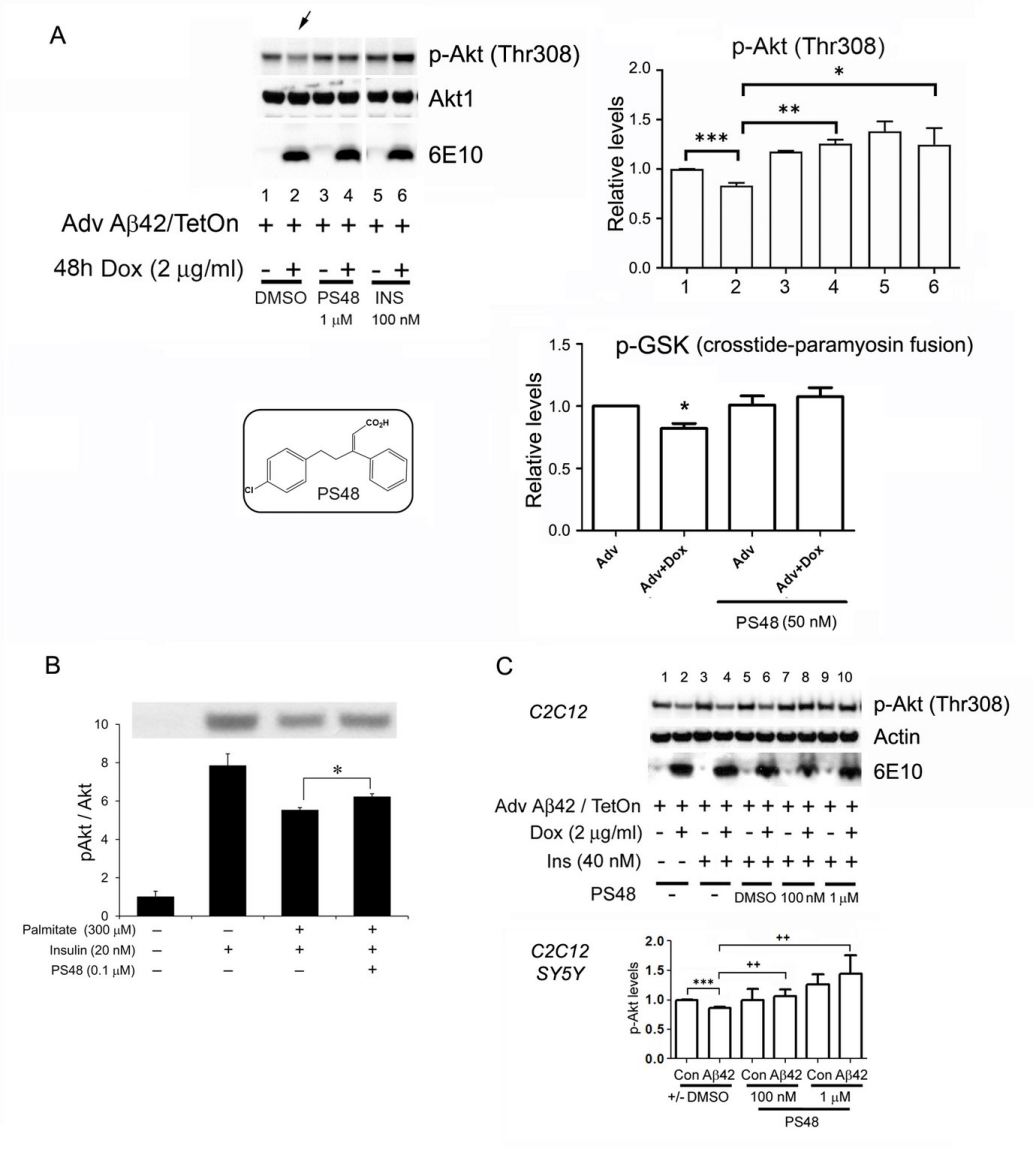
PS48 or Insulin restores Akt activation in β-amyloid expressing cells. **1A, left**. SH-SY5Y cultures were infected with Adenovirus (Adv) encoding Aβ42 (24–36 hrs) and induced with Doxycycline (46 additional hrs). Amyloid bearing cells show inhibited Akt phosphorylation (lane 2 arrow). PS48 (1 μM) or high dose Insulin (100 nM) added 2 hrs before doxycycline induction (pre-treatment) restores Akt activation levels in cells expressing Aβ42 (lane 4 and 6). Low dose Insulin 40 nM (stimulation) is added to all cultures 20 min prior to harvest. PS48 alone does not over-stimulate Akt (lane 3 vs. 1). **1A, right**. Quantification of restored inhibited Akt activation by PS48 and Insulin. pT308 Akt levels are normalized RFU, (relative fluorescence units). n = 3 experiments. Error bars are 1 SE relative to lane 1 (control). Brackets indicate individual t-tests comparing Aβ expression result (lane/bar2) to: control (lane/bar1), PS48 (lane/bar 4) and Insulin (lane/bar 6) additions. *** p < .001, **p = .01,* p < .05, t-test. Insulin increases pAkt T308 over control (p = .02, lane/bar 5 vs.1). PS48 also has a stimulatory effect (p < .01, lane/bar 3 vs.1). ANOVA 1-way: P = .03, F3.7; 2-way: P = .06, F = 3.1(between groups), P = .49 (within treatments, ie. replicates). **1A**,**below**, *in vivo-in vitro* coupled assay of Akt activity. Akt was immunoprecipitated from SH-SY5Y cells infected with Adv. Phosphorylation of GSK3α/β consensus peptide proceeded *in vitro* after adding 200 μM ATP to the immunoprecipitate. PS48 pre-treatment (50 nM), added to cells 2 hrs. before Doxycycline addition, prevented the inhibtion of peptide phosphorylation by Aβ42. (Western not shown, n = 3, *p< .05 vs. control (Adv. alone). PS48 structure, left. **1B**. Non-amyloid-based insulin resistance model. Primary rat cortical neurons (PCN) exposed to fatty acid neurotoxin, palmitate (300 μM). PS48 (100 nM, 24 hrs) partly reversed inhibition of Akt Ser473 phosphorylation by palmitate, in insulin-stimulated cells (20nM, 15 mins before lysis). Identical result in ceramde (50 μM)-treated PCNs, not shown). (*p < .05, n = 3). **1C.** SH-SY5Y and C_2_C_12_ cells (representative Western) were used to test dose dependency of PS48 (1 μM, 100 nM, 10 nM). Aβ42 expression inhibits the insulin-stimulated phosphorylation (activation) of Akt (lanes 4 vs.3 and 6 vs.5 were combined to correspond to bar 2 vs.1). PS48-, doxycycline-, Insulin- additions and harvest as above. Data from both cell types were combined for the bar graph. PS48 100 nM and 1 μM (10 μM, not shown) each significantly overcome the Aβ42 effect (lanes 8 and 10 vs. 4 or 6). Bar graph: *** p < .005, Aβ vs Con; **++** p < .01, PS48/Aβ (bars 4 and 6) vs. vehicle/Aβ42 (bar 2). DMSO is vehicle control. (n = 3).

The pathological target identified in these *in vivo* and *in vitro* platforms suggested that a small molecule could be found that modulates the insulin-PDK-Akt activation cycle in such a way to relieve the inhibitory amyloid effect. A chemical data base search identified an allosteric activator of PDK-1 (CAS 1180676-32-7, PS48), a chlorophenyl pentenoic acid having a MW of 286.7 [106] (**Fig 1 top**). Furthermore, it has an inactive ‘E’ isomer, PS47, for control use [96, 97]. It is unique in its action to bind the hydrophobic motif/PIF binding pocket of PDK and not the ATP binding site. The compound has other possible beneficial actions that may translate to improve hippocampal neurogenesis [107].

Employing the above assays, the SHSY5Y neuroblastoma line was used for most cell-based experiments. First, to model insulin resistance in these cells, Akt (PKBα) activation with increasing insulin concentrations applied shortly before harvest was established (**S1A Fig in S1 File)**. Then at the same doses, cellular expression of Aβ42 over the preceding 48 hours was introduced to produce insulin resistance. At moderate insulin doses (20 nM), Akt activation is inhibited by Aβ42, whereas high doses of insulin (100 nM) overcame the effect. Thus Aβ42 was shown to desensitize insulin action, raising the insulin concentraton threshold to achieve an equivalent response (**S1B Fig in S1 File)**. In this system, we find that PS48 exposure did not intrinsically activate basal Akt. However, in the presence of low dose insulin (3 nM) where Akt activation is subthreshold, PS48 augmented the response. This is in line with its purported allosteric action to positively modulate PDK activity (**S1C Fig in S1 File**). At moderate insulin doses, that produce robust Akt phosphorylation, PS48 did not further enhance it. Neither did PS48 intrinsically affect at least one critical downstream factor in this pathway, mTOR(**S1D Fig in S1 File**). These properties make it ideal to test if it will protect insulin signaling against amyloid peptide toxicity, while not over-regulating the pathway.

First we tested PS48 (1 μM) in cultured SH-SY5Y cells, where it is shown to normalize β-amyloid expression-induced inhibition of Akt activation (pT308 phosphoryation) by subacute moderate dose insulin (40 nM) applied 2 hours before harvest (**Fig 1A**). Notably, it did not overactivate basal levels of phosphoAkt to a statistically significant extent (lane/bar 3 vs 1). Live cells were pre-treated with either PS48 (1 μM) or high dose Insulin (100 nM) for 2 hrs. before doxycycline was added. Aβ42 expression proceeded over the next 46 hrs. PS48 and high dose insulin had similar protective actions for the duration of expression. pAkt levels were quantified densitometrically, shown right. Aβ expression at moderate toxic levels to the cell, reduced Akt phosphorylation with an effect size of -0.17±0.02 relative units (p < .001 t-test; mean difference of 1.00–0.83±0.03 RU). PS48 addition recovered pAkt to control levels with an effect size of 0.42±0.04 RU (p < .01 vs. Aβ expression; mean difference of 1.25± 0.04–0.83), as did insulin (effect size 0.41±0.19; p < .05; mean difference of 1.24± 0.17–0.83; lane/bars 4 or 6 vs. 2). ANOVA yielded P = .03, F = 3.7 (1-way); P = .06, F = 3.1 (2-way). To confirm, we next tested Akt enzymatic activity to phosphorylate a consensus substrate peptide corresponding to phopho-Ser21/9 sites on GSK3-α/β. We obtained the same outcome, mainly that lower dose PS48 (50 nM) also protected Akt activity from inhibitory cellular Aβ42 expression. We also tested the effect of PS48 to reverse a non-amyloid dependent model of insulin pathway toxicity, exposure to the long-chain saturated fatty acid, palmitate, in rat primary cortical neurons. In **Fig 1B**, insulin (20–40 nM) given just 20 min prior to harvest, stimulates Akt phosphorylation, which palmitate (300 μM) partly blocked (~30%). PS48 significantly corrects some of this inhibition. In **Fig 1C**, we confirm and expand results of 1A, testing various doses of PS48; 100 nM, 1 μM, (10 μM not shown) in both SH-SY5Y and C_2_C_12_ myotube cell lines. A dose dependent effect to reverse Aβ42- provoked inhibition of insulin-stimulated Akt phosphorylation became apparent at 100nM. Insulin-stimulated pAkt levels were reduced from baseline in the presence of Aβ expression with an effect size of -0.14±0.09 relative units (p < .001; mean difference of 1.00–0.85±0.03 RU). 100 nM PS48 recovered pAkt to control levels with an effect size of 0.20±0.15 RU (p < .01 vs. Aβ expression), as did 1 μM PS48 (effect size 0.58±0.13; p < .01 t-test and p = .0003 Dunnett’s. ANOVA yielded P = .0013, F = 5.1 (1-way); P = .013, F = 3.8 (2-way).

Several downstream effectors and substrates of Akt were examined for sensitivity to Aβ toxicity and PS48. CREB, the cAMP response element binding transcription factor, has pleiotropic actions to promote neuronal survival, progenitor proliferation, neurite outgrowth and differentiation. It is also well documented to control the activity-driven and neurotrophin-dependent expression of proteins essential to long term memory formation (LTM) and synaptic plasticity (LTP) (see reviews by [42, 108, 109]). It is situated in the PI3K/Akt/CREB pathway to transduce effects of Insulin, IGF-1 and BDNF on protein expression critical to neurogenesis and plasticity [40, 43, 110]. CREB supports LTM by stabilizing synaptic strength, regulating intrinsic neuronal excitability and recruiting subsets of neurons in the hippocampus and amygdala that encode the memory trace [111–114]. We focused on CREB because it can be directly activated by Akt [115, 116], is protective against neuronal apoptosis [117, 118] and supports LTP [119]. We found consistent inhibition of insulin-stimulated CREB phosphorylation (pS133) by intracellular Aβ42 and this was also stabilized by PS48 (50 nM) (**S2A Fig in S1 File**).

Previous work had shown sensitivity of endogenous GSK3α/β (inhibitory S9A phosphorylation) to viral expressed Aβ42 [80], however the current experiments under combined Aβ42 pressure and PS48 proved inconclusive. Nevertheless, PS48 had no effect on resting cellular pGSK levels (**S2B Fig in S1 File**). Finally, in testing for changes in activating phospho-levels of indirect downstream substrate and metabolic sensor mTOR, we found that neither Aβ expression (also shown in [105]) nor PS48 applications had any effect (**S2C Fig in S1 File**).

**Fig 2:**
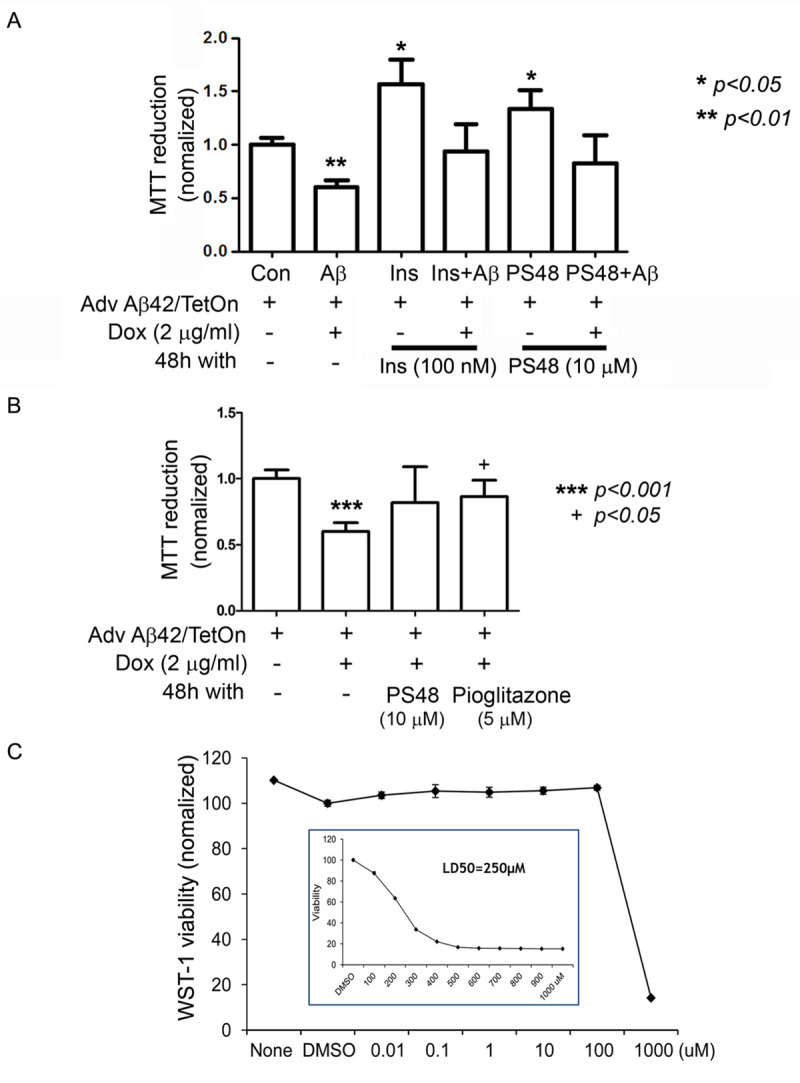
PS48 or Insulin partly restores partial viability in β-amyloid expressing cells. **2A**. SH-SY5Y cultures infected with Adenovirus encoding Aβ42 and induced with Doxycycline. Amyloid bearing cells are less viable in an MTT reduction assay (bar 2 vs. 1; t-test, p < .01). Pretreatment with insulin or PS48 partially reverses cell death to control viability levels (bars 4 and 6 compared to Con). Insulin or PS48 alone are also neurotrophic (bars 3 and 5 compared to Con, * p <05, n = 3). ANOVA 1-way: P = .003; 2-way P < .0001 (column effect). See text for treatment effect sizes (given as mean differences). **2B**. In C_2_C_12_ myotubes, 48 hrs pretreatment with PS48 (10 μM) is comparable to pioglitazone (5 μM). +p < .05 vs. bar 2. **2C**. Using a WST viability assay, cultured N2a cells are unaffected by PS48 at doses up to 100 μM, estimated LD50 is 250 μM. 24 hrs. exposure, n = 4.

In parallel experiments, PS48 pre-treatment partially restored the Aβ-induced decrement in cell viability. (**Fig 2A**, MTT reduction to formazan assay). SH-SY5Y cells were exposed to Adv TRE-Aβ and ± doxycycline to induce Aβ42, then harvested at 48 hrs. Amyloid bearing cells are less viable compared to control (bar 2 vs. control bar 1; t-test ** p < .01; effect size (mean difference) = -0.41, confidence intervals [-0.20 to -0.62]. Pretreatment of live cell cultures (before adding doxycycline) with high dose insulin (100 nM) or PS48 (10 μM) reverts cells in the direction of normal (basal) conditions (bars 4 and 6 vs. con bar 1 were not significantly different). However, they were also not significantly different from Aβ-laden cells (bar 2). Effect sizes (and 95% CI) for Ins+Aβ vs. Aβ and for PS48+Aβ vs. Aβ are 0.33 [-0.12 to 0.79] and 0.22 [-0.27 to 0.71], respectively (ANOVA ns). We therefore conclude a trend effect. Nevertheless, in the absence of Aβ expression, both Insulin and PS48 appear to have significant trophic effects (bars 3 and 5 vs. con bar 1; * p < .05). Overall, ANOVA yielded a P = .003 (1-way) and P < .0001 (2-way). The same cytoprotection was obtained in C_2_C_12_ myotubes. In **Fig 2B**, PS48 also compared favorably with a PPAR agonist, pioglitazone. The toxicity profile of PS48 itself was determined in a similar reduction assay (WST-1) and is well tolerated by cells (**Fig 2C**, LD50 250 μM).

**Fig 3:**
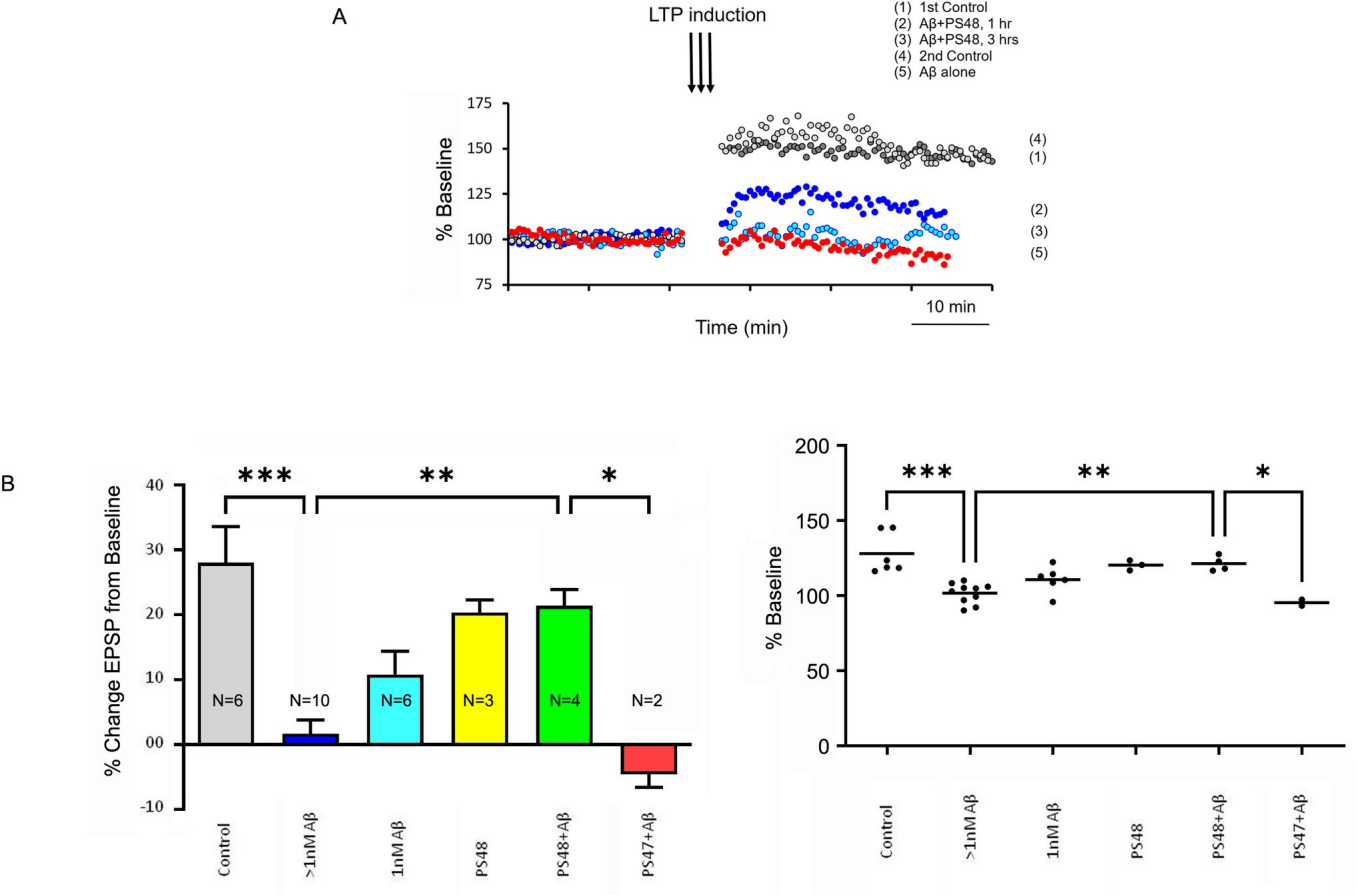
PS48 partly restores the suppression of LTP by Aβ42. **A**. Day 14 rat prefrontal cortex (PFC) exposed to Aβ oligomers (0.5 μM) or Aβ plus PS48 (10 μM). Superimposed representative single recordings made under conditions 1 through 5, as noted. Control is DMSO-vehicle. EPSPs recorded from layer 2; stimuli (32 μA = ½ max ampl., 2 Hz) applied to layer 5. Aβ completely suppressed LTP (5 vs. 1). PS48 in the prsence of Aβ restored 68 ± 18% of control potentiated EPSP (1 hour perfusion, condition 2). A 3 hour application however showed near complete run-down (3). Aβ and Aβ/PS48 experiments were restored to control LTP levels after washout (4). **B**. Quantification of LTP data. Bar graph (left). LTP induction shown as mean % change (increase or decrease) from baseline (± SEM). n independent replicates are noted. Scatter graph (right) gives means of individual experiments and group averages as unadjusted % baseline values (normalized at 100%). All concentrations Aβ> 1 nM (high dose, 2.5–500 nM) completely abrogated LTP. PS47 is an inactive isomer control (n = 2). n replicates as shown. *** p < .0001 Aβ alone (high dose) vs control, ** p < .01 PS48 treatment vs Aβ alone (high doses), * p < .05 PS48 treatment vs. P47 control (Aβ high doses); ANOVA and Tukey’s tests (see text for additional statistical details).

PS48 was further tested *in vivo* and found to partially reverse the inhibition of long term potentiation (LTP) caused by added oligomers of synthetic Aβ42 peptide (**Fig 3**). Acute prefrontal rat cortical slices were super-perfused with Aβ42 peptide (0.5 μM) prepared as amyloid diffusible ligands (ADDLs), shown to be largely comprised of oligomeric species. LTP was measured as % baseline EPSP amplitudes. Representative single experiments showing EPSP amplitudes (expressed as % baseline) under the following conditions: control LTP (1), inhibition after Aβ 5 nM (5), Aβ plus PS48 10 μM after 1 (2) and 3 hrs. (3) exposure (showing a run-down effect) and reversibility to control LTP after washout (4), are superimposed in **Fig 3A**. Quantification of data from n = 3 to 10 independent experiments is given in chart (mean of means as EPSP % change above baseline) and scatter (individual means comprising the group mean, as EPSP % baseline) graphic forms, respectively (**Fig 3B)**. LTP was completely abrogated by all concentrations of Aβ42 > 1nM (2.5–500 nM) (***p < .0001, 1-way Tukey’s multiple comparisons), whereas Aβ at 1 nM partially did so (p < .05, Tukey’s). 10 μM PS48 alone produced a small, nonsignificant decrement in mean LTP compared to control. However, in the presence of higher dose Aβ (>1 nM), this level of LTP was sustained by PS48, representing a significant improvement (bar 5 vs. 2, **p < .01, Tukey’s). For instance, the mean effect size of PS48 (10 μM) to improve LTP induction at a fixed Aβ concentration of 5 nM, is a net 18% ([95% CI; 27.1 to 8.9%], t-test p = .0065, n = 3 and 4 respectively). The control inactive stereoisomer PS47 provided no benefit compared to PS48 in the presence of Aβ (*p < .05 bar 4 vs. 5, Tukey’s), in fact slightly accentuated the Aβ effect. ANOVA results: 1-way: P < .0001, F = 10.2; 2-way P = .0018 for group treatments, P = 0.8 for row (within group) effect.

**Fig 4:**
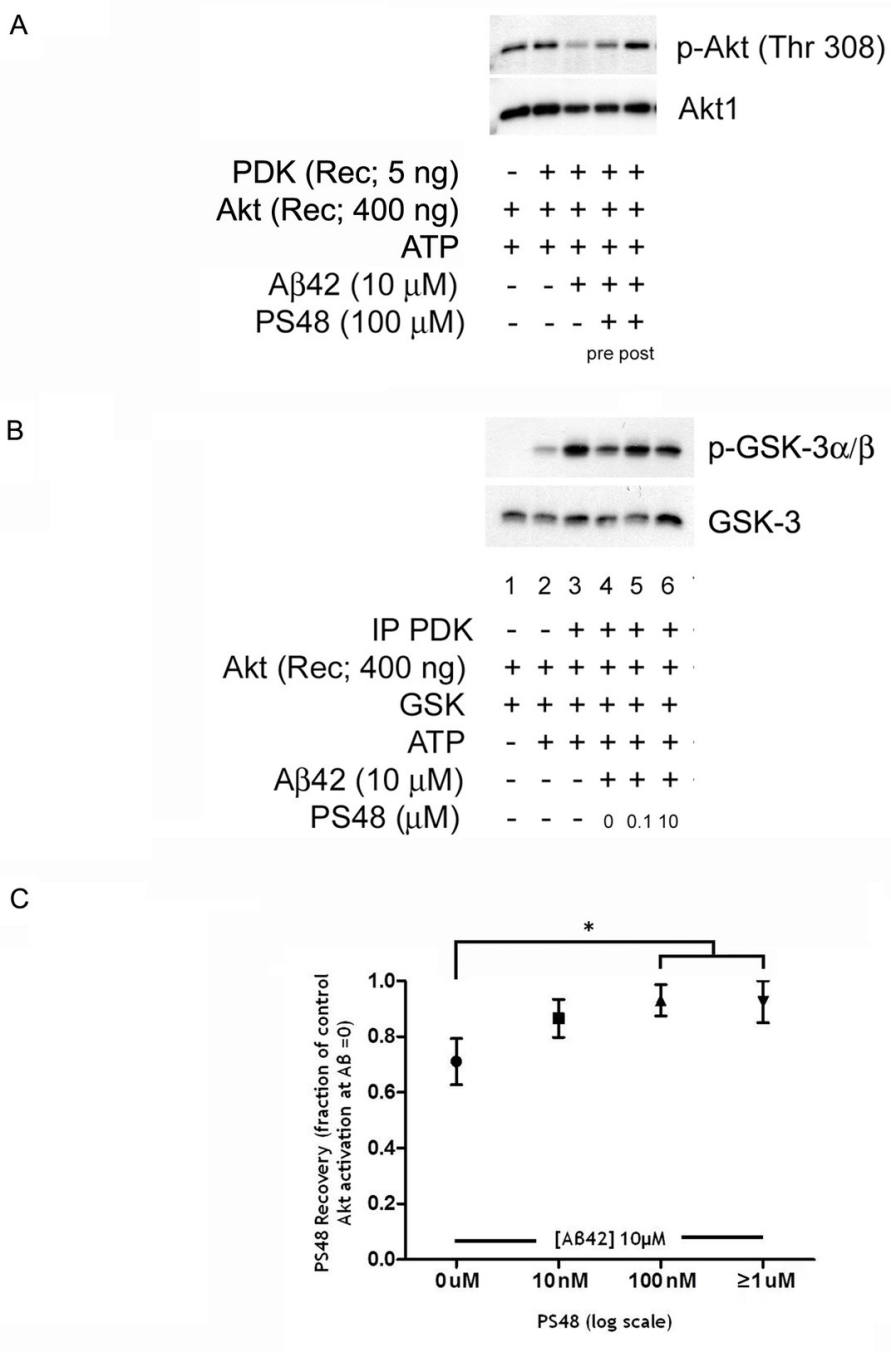
PS48 partly restores inhibited Akt phosphorylation (Akt activation) and GSK3β phosphorylation (Akt activity). In vitro additions of recombinant PDK (5 ng; **4A**.) or pre-immunoprecipitated PDK-1 (**4B**.). **4A**. Aβ peptide addition near completely results in Akt dephosphorylation (lane 3). PS48 added after Aβ peptide (post) in the reaction mixture completely reverses the loss of Akt phosphorylation (lane 5 vs. 3). When pre-added (pre), PS48 partially restores pAkt levels (lane 4). Note commercial recombinant Akt lots come variably phosphorylated (lane 1), but lack enzymatic activity in control reactions until PDK is added (see Figs 4B and 6B and text) and also undergo further phosphorylation. **4B**. Restoration of Akt-catalyzed phosphorylation of GSK3β peptide (lanes 5 and 6 vs. 4). **4C**. Quantified trend to reverse inhibited Akt activation and activity by PS48 in the presence of 10 μM Aβ42 oligomers (ADDLs). PS48 is active in the 10 nM range. Data from pAktT308, pAktS473 and pGSK3α/β S9 experiments were normalized and pooled (n = 3–6 ea.). ’Fraction of control Akt activation’ is the [Aβ (± PS48 presence) /Control] densitometry signals ratio, where 1.0 represents full restoration.* p < .02, t-test, Aβ 10μM/0μM PS48 (n = 6) vs. combined 100 nM and ≥ 1 μM (1, 10 and 100 μM) data (n = 8). The mean difference is 0.23 ± 0.08, 95% CI = [0.41 to 0.05].

We next tested PS48 in an *in vitro* assay of both Akt activation (phosphorylation of T308) and enzyme activity. In **Fig 4A**, recombinant Akt and PDK-1 were added to a reaction mixture containing synthetic Aβ42 peptide oligomers (10 μM) and ATP to start the reaction. Some experiments employed added PI3P and/or pre-dephosphorylation of Akt by treatment using PP2A, with variable improvements in the efficiency of activation. A GSK fusion peptide was added as substrate for the enzymatic readout (Westerns of phospho-S9 GSK3α/β). Initial experiments used high dose PS48 (100 μM) and tested if added before (pre) or after (post) the Aβ peptide made a difference. pAkt-T308 levels were reduced in the presence of Aβ, similar to the cell-based expeiments. PS48 restored Akt phosphorylation. Moreover, it had greater efficacy when added after Aβ peptide equilibration. So, this procedure was followed in **Fig 4B**, that alternatively used recombinant Akt and immunoprecipitated PDK-1. PS48 was found to be active at 0.1 and 10 μM in reversing the inhibition of GSK phosphorylation. The pooled results from same experiments over a range of PS48 concentrations are quantified in **Fig 4C**. In the presence of 10 μM Aβ42, ~30% of Akt activation is inhibited (expressed as a remaining fraction of the control (absent Aβ, set to 1.0), thus 0.70 ± 0.09. Beginning at 10nM PS48, activation/activity is increasingly normalized until a maximum of 0.95 ± 0.08 of control is reached at ≥1 μM PS48 (includes 10 and 100 μM data points). When all values ≥ 0.1 μM are combined (n = 8), the trend toward normalization reached significance (p < .02, compared to 0 nM PS48, t-test, n = 6).

**Fig 5:**
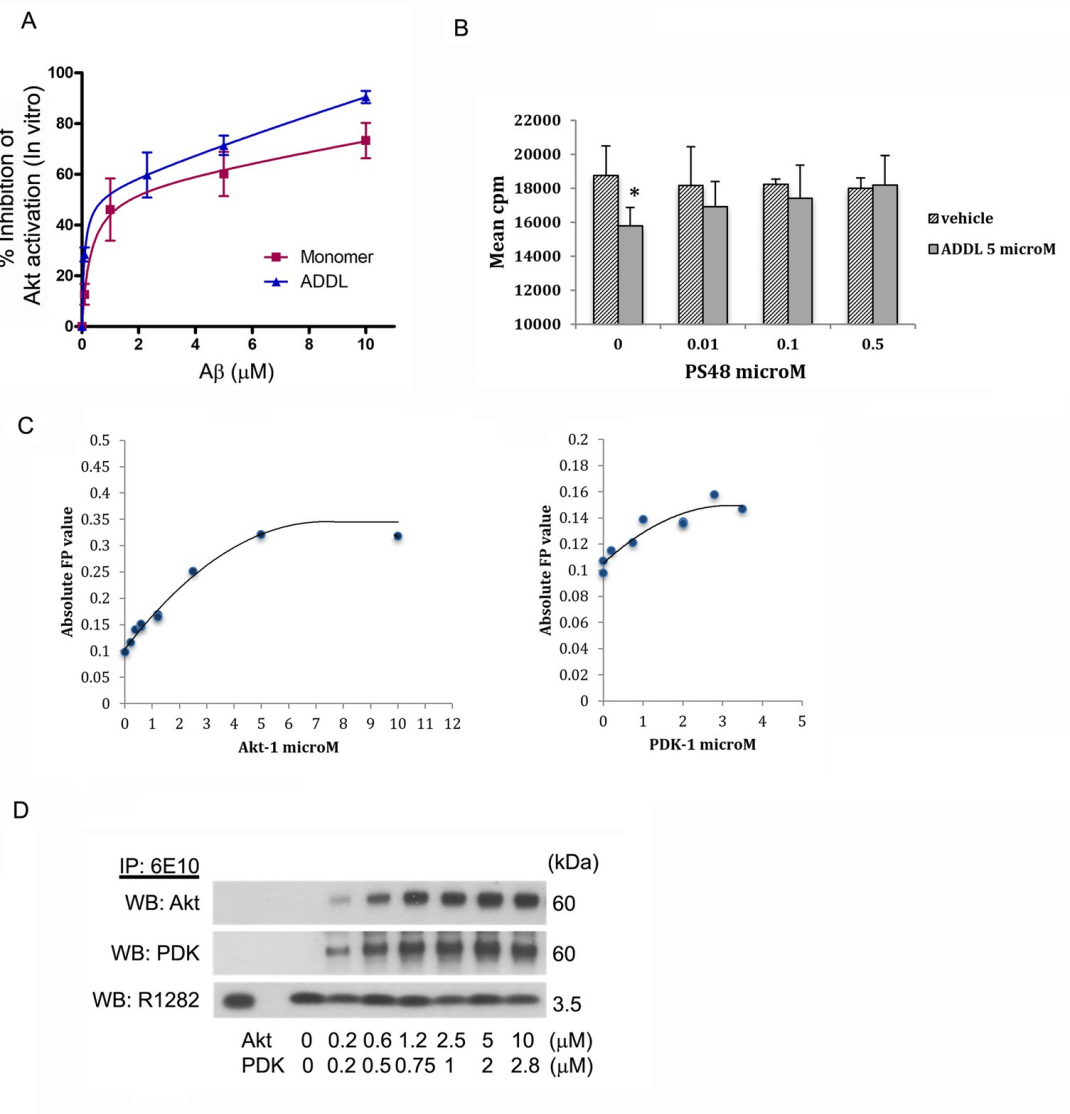
Equilibrium enzymatic and binding studies. PDK-1 and Akt-1 are Aβ42 targets. **5A**. Aβ42 monomers and ADDL-oligomer preparations, at concentrations shown, exhibit saturation effects to inhibit the activation of Akt by PDK-1. In vitro assay data are presented as % inhibition of either activation of Akt (phospho-T308) by PDK-1 or of its subsequent activity to phosphorylate a GSK3β consensus peptide fused to paramyosin (’crosstide’). Densitometry results from both western blots were combined. Imax ~ 52% for both Aβ preparations: ADDL, K0.5 = 0.08 μM; monomers, K0.5 = 0.31 μM. n = 2–7 experiments each point, ± 1 SEM. **5B.** In vitro radioassay (see methods). PS48 from 10 nM to 0.5 μM, gradually diminishes the inhibitory effect of Aβ (5 μM ADDL alone, bar 2, *p < .05 vs. vehicle) on the phosphorylation of GSK peptide (measured in cpm). The trend is toward control activity, not significant from bar 1 or other vehicle controls. **5C**. Quantification of Aβ42 binding to kinase targets by fluorescence polarization (FP). The probe was FAM-tagged-Aβ42. Recombinant kinase titrations shown along bottom. The spectrophotometric polarization signal increases as the probe becomes more restricted by receptor binding. Bmax: Akt 0.32; PDK 0.16. IC50: Akt ~2 μM, PDK ~1 μM. **5D**. Direct binding of recombinant PDK-1 and Akt-1 to tagged Aβ42 in solution is confirmed and exhibits saturation characteristics. Aβ42 was immunoprecipitated (6E10), fractionated by Western and co-precipitates are detected using anti- Akt and -PDK. Aβ42 concentration was fixed at 200 nM and detected using polyclonal R1282. Lane 1 is Aβ peptide control, lane 2 is beads without 6E10 control.

To further explore target engagement, we probed the characteristics of Aβ42 interaction with PDK-1 and/or Akt-1. We conducted equilibrium Aβ42 dosage and binding experiments in solution to the respective kinase targets. First, the *in vitro* pardigm above was used to test whether Aβ42 peptide exhibits a dose-dependant specificity to inhibit the activation (phosphorylation of T308) and enzymatic activity of Akt (to phosphorylate its consensus substrate). The combined Western densitometry data in **Fig 5A** suggests the possibility that either Aβ monomers or oligomers act to inhibit either one or both of the two kinases, separately or in complex, and in a saturable manner. Using a 2 site, non-linear fit algorithim a Imax value of ~ 52% inhibition of control activation is obtained for both species, however the oligomers showed greater affinity (ADDLs: K0.5 = 0.08 μM, monomers: K0.5 = 0.31). To independently confirm the reversal of the PDK/Akt activation sequence by Aβ42 peptide, we employed a modified radiolabelled-based assay in **Fig 5B**. Again, PS48 dose-dependently reduced the inhibition of P^32^ -labelled phosphate addition to a consensus peptide. The effect was first noticed at 10 nM (**Fig 5B**), consistent with Fig 4C. Next, a novel assay was adapted to determine if Aβ42 bound to either PDK-1 or Akt-1 in solution and to discern any effect of PS48 on this, using the fluorescence polarization (FP) technique. A spectrophotometric filter detects the fluorescent signal from a probe that becomes polarized once restricted by receptor binding. We found that the FP signal increases as the concentration of either recombinant PDK or Akt are increased. The probe concentration (FAM tagged-Aβ = 200 nM) is fixed in this procedure. Results shown are from 2 experiments each. Saturable binding to Aβ is shown by both target molecules, where the Kd (1/2 max constant) for PDK shows slightly increased affinity over Akt, but fewer binding sites (**Fig 5C**). PS48 additions did not affect either polarization signals, concluding that Aβ42 is probably not competitively occupying the pocket site (results not shown). The in-solution FP assay was validated by co-immunoprecipitating the bound reaction products. IP of FAM-labeled Aβ42 with anti-6E10 pulls down increasing amounts of Akt and PDK according to the titration until saturation (**Fig 5D**). Aβ concentrations were constant as shown in the anti-R1282-developed blot shown below. Previous work has shown that Aβ peptide in AD brain co-immunoprecipitated with PDK and Akt and that cellular expression of Aβ42 disrupts Akt-PDK interaction [64].

**Fig 6:**
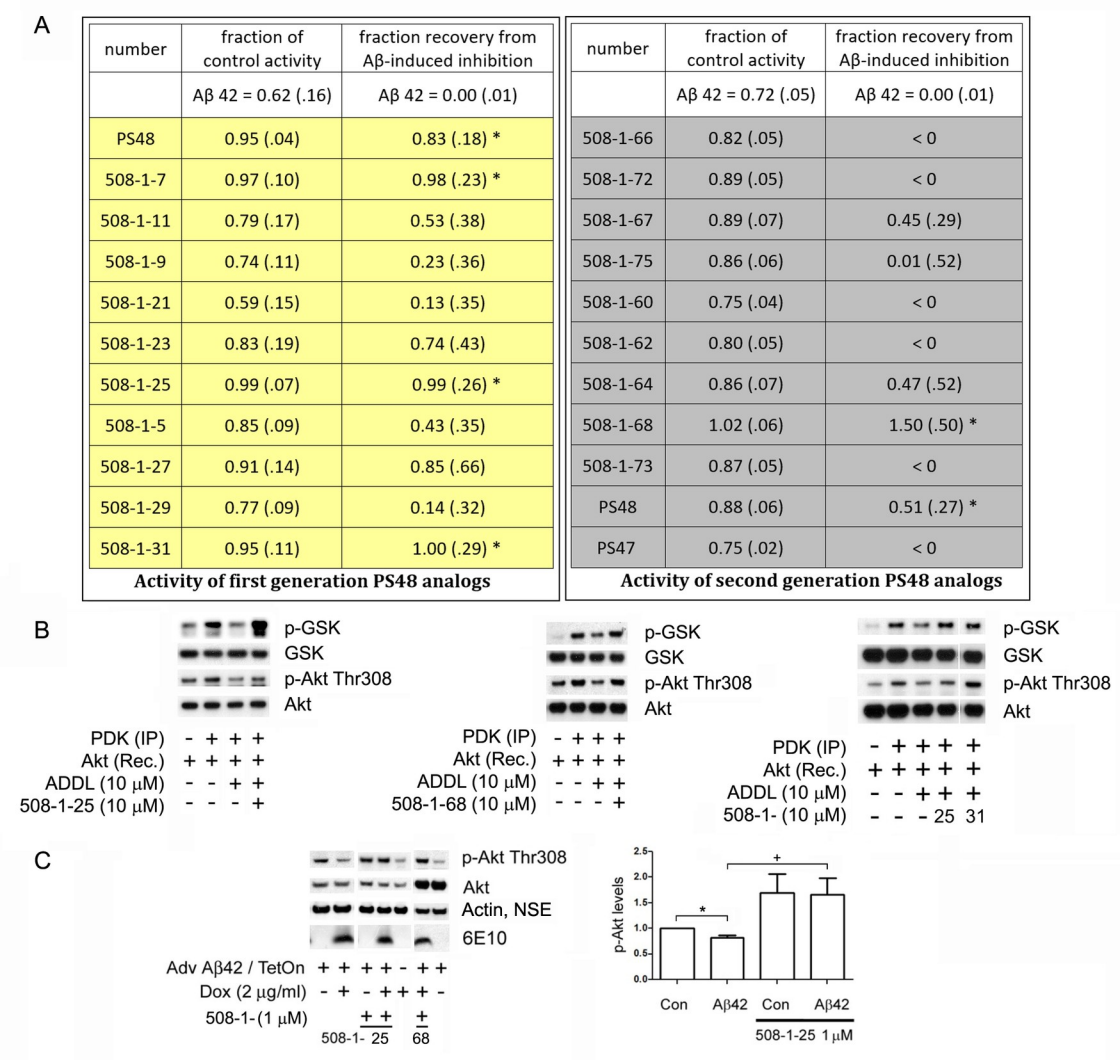
In vitro screen of focused compound library based on PS48 scaffold. **6A**. Compounds numbered according to SAR. Left, first generation; Right, second generation compounds. Two indices of effectiveness are tabeled: ’Fraction of control activity’ represents the observed amount (or fraction) of activation of Akt by PDK in the presence of compound with amyloid, relative to the no drug, no amyloid control (full activity = 1.00, Aβ42 alone is 0.62). ’Fraction of recovery’ is an index measure of the ability of a compound to restore the activation of Akt by PDK in the presence of Aβ, relative to the level of inhibition from Aβalone (drug-Aβ)/(control-Aβ) signals ratio. (full activity = 1.00, Aβ42 alone is 0.0). * p< 0.01 for both measures, n = 5 independent experiments each compound. PS47 is the inactive stereoisomer of PS48, bottom right. Final concentrations of both Aβ (ADDLs) and drug were 10 μM. Results are combined phosphorylations of Akt (T308, Akt activation) and pGSK3β consensus peptide substrate (S21/9, Akt enzymatic activity). **6B.** Selected Westerns of in vitro reaction mixture by-products, highlighting significant GSK peptide (S21/9) and Akt (T308) phosphorylations in the presence of Aβ by compounds 508-1-25 and -31 (1st generation) and 508-1-68 (2nd generation). These ’hits’ restored activations at or better than PS48. **6C**. In vivo verfication studies in SH-SY5Y cells. Addition of Doxycycline results in intracellular amyloid accumulation (6E10 blots). Compounds 25 and 68 (1 μM) intrinsically boost Akt phosphorylation to similar degrees, that remain sustained under Aβ pressure (as the case with PS48). Phospho-Akt levels, both T308 (as exampled) and S473 (not shown) were quantified and pooled for the bar graph, featuring 508-1-25. All cultures were stimulated with insulin (40 nM, 240 ng/ml) in the last 20 minutes of the Aβ- expression period. n = 3 experiments. * p < .05 vs. Con, **+** p < .01 vs. Aβ42.

Lastly, we launched drug screening experiments using a focused library of novel compounds that were synthesized using PS48 as the starting scaffold. Two generations were created based on structure-activity relations (SAR) in an attempt to optimize performance. The quantified results of western-based *in vitro* assays and signal intensities, compared to PS48, are shown in **Fig 6A.** The activity of each compound against the Aβ42 effect to inhibit the PDK-Akt activation sequence is shown as two indices: 1) ’fraction of control activity’, as remaining in the presence of Aβ with drug [Aβ with drug]/[control], where the control is absent Aβ and normalized to 1.00. The fraction of control activity observed in reactions having just Aβ alone (maximal inhibition, no drug) was 0.62 ± 0.16 (top left), and 2) ’fraction recovery to control level from Aβ42-induced inhibition’, where the index is normalized instead to the Aβ inhibitory effect size [Aβ with drug—Aβ]/ [control—Aβ] signals. The fraction recovery with just Aβ alone (or no drug effect) is therefore 0.0 on this scale (top right). Using either index, a value of ≥1.00 is a complete reversal. Based on this data, a rough SAR is beginning to emerge where it appears that the greatest effect is realized by modifying the acidic portion of PS48, e.g. extension from the linker and aryl groups. An increase in potency over PS48 was achieved with compounds 7, 25, 31 and 68. Examples of *in vitro* assay performance are shown below (**Fig 6B**) for compounds no. 25 and 31 (generation 1) and 68 (generation 2). These were then validated as correcting the inhibition of Akt phosphorylation in the cell-based model of Aβ toxicity (as in Fig 1), shown at the bottom for compounds 25 (with quantification) and 68 (**Fig 6C**). The control stereoisomer PS47 had no effect in these *in vitro* reactions (table bottom right). The new ’hit’ compounds also had no direct or indirect effects on potential downstream off-targets such as mTOR (protein levels and phosphorylation status, results not shown). Moreover, the LD50 of several promising compounds in N2A neural cultures proved even higher than PS48 (e.g. no. 25; 350 μM, WST assay, result not shown).

## Discussion

Akt (PKB) is an essential kinase in the insulin/IGF signal cascade having pleotropic influence over many cell survival and metabolic pathways. It’s co-crystal structure in complex with a substrate peptide (GSK3β) and an ATP analog, reveals the structural relationship between the C-terminal hydrophobic motif (HM) and the activating phosphorylation of Akt on the Thr 308 residue by PDK-1 [120]. The co-crystal structure of PDK-1 in complex with ATP reveals the HM-binding pocket (PIF domain located on the N terminus) and phosphoSer- binding pocket through which it docks with its many substrates including: Akt, SGK, S6K, PKC and RSK [121]. Interestingly, Akt is the only substrate not requiring docking at the PIF-pocket site to undergo catalytic activation by PDK-1 [122, 123]. PS48 and family of it’s analogs are small molecule allosteric activators of PDK-1, binding within the PIF pocket [97], thereby facilitating Akt activation by IGF-1 [96, 106, 124]; for review see Xu et al. [125]. These features are presented in schematic in **S3 Fig in S1 File**. Additional actions of this allosteric binding include supporting the induction of pluripotent stem cells from somatic cells [107].

Herein, we characterized PS48 action in the context of insulin signaling using neuroblastoma cell lines and then demonstrated its ability to partially or wholly normalize Aβ42 oligomer induced insulin resistance and toxicity using an adenoviral expression system. First, a dose dependency of Akt activation by insulin in PCN and N2a cultures was established. Intracelluar expression of Aβ42 oligomers resulted in a reduction of sensitivity to insulin, such that higher insulin doses were required to overcome the resistance. At low, subthreshold doses of insulin (3 nM), PS48 pretreatment appeared to sensitize Akt activation. Next, PS48 (0.1 to 1 μM) is shown to significantly counter the inhibitory effect of Aβi expression on submaximal insulin-induced Akt phosphorylation in live cells, similar to the effect of a higher insulin dose (100 nM) alone. The same findings were obtained in another insulin responsive cell line, C2 myotubes. Moreover, PS48 partially overcame insulin resistance in a non-amyloid model of cellular toxicity, to the saturated fatty acid palmitate. A downstream effector and substrate of Akt, CREB, is also hypophosphorylated after Aβ expression, and accordingly corrected by treatment with PS48. On the other hand, another effector, phospho-mTOR, remained unaffected by either treatment. The lead compound furthermore partially prevented Aβ-induced cell death in a neuroblastoma cell line, as did high dose insulin and pioglitazone treatments. Next, it significantly normalized the effect of synthetic Aβ peptide (ADDL oligomers) to inhibit LTP in rat prefrontal cortical slices. To test the purported cellular step involved in this mechanism of insulin resistance, we performed *in vitro* reactions using recombinant Akt and constitutive active PDK kinases to phosphorylate a GSK3α/β-based consensus peptide substrate. Aβ oligomers (ADDLs) inhibit Akt activation and crosstide phosphorylation and PS48 (10 nM to 1 μM) restored this to ~90% of control levels. Mechanistically, tagged Aβ42 is shown to bind to both recombinant Akt and PDK using an in-solution fluorescence polarization assay, the two kinases having different affinities and saturation levels. Finally, *in vitro* and cell-based assay platforms were employed in a focused medicinal chemistry effort to probe the structure-activity characteristics of the parent molecule. Other analogs were found that reversed the inhibited Akt activity by better than 90%.

The two main physiological readouts of Aβi toxicity in this study that were partially protected by PS48 were cell death and inhibited synaptic plasticity (LTP). We show that PS48 and analogs in development significantly improved Akt activation by insulin from inhibition by Aβi accumulation. Akt is critical to both neuronal survival [126] and LTP as demonstrated in prefrontal cortex, amygdala and hippocampus, [127–129]. Hippocampal LTP is particularly sensitive to Aβ oligomers [48, 49]. Among the possible effectors of these two outcomes, we find the CREB link plausible because this result mirrored the Akt responses to Aβi and PS48 (Fig 1). CREB is activated by several canonical receptor-activated kinase pathways (i.e. PKA, CaMK, MAPK). In particular, BDNF/TrkB receptor activation has been well studied [130–132]. However, insulin and IGF-1 also phosphorylate CREB via PI3K/Akt [43], and this can occur directly (see also [133, 134]). Another effector intervening between PI3K/PDK/Akt and CREB is GSK3αβ. Akt stimulation results in GSK3 inactivation, resulting in CREB de-repression (via inhibitory pS129: [135, 136]). Moreover, GSK3 activation promotes apoptosis [137, 138] and depresses spatial learning and LTP in mice [139, 140]. Although our results were inconclusive on cellular GSK, the Akt activity assay data using GSKtide could still be consistent with a partial role for this mechanism in our endpoints.

Several limitations of this work will require clarification in future studies. Practical ones include finding the optimum concentrations of drug to test, which depends on the assay employed. Taking for instance *in vitro* drug screening, the optimum may actually lie between 100 nM and the 10 μM concentrations reported here (Figs 4C and 6). The same applies to cell viability assays (Fig 2). Another issue that arose during the *in vitro* reactions was the basal phosphorylation status of commercial, recombinant Akt. It was generally high and only partially decreased by PP2A treatment. However, we believe this does not affect our results because drug efficacy was based on: 1. maximal stimulation with PDK (recomb Akt was always further phosphorylated after PDK (constitutively active) addition, regardless of its basal phospho state), 2. the level to which Aβ inhibited this, and 3. the recovery from that by drug. Moreover, regardless the phospho level of unstimulated recombinant Akt, it had no enzymatic activity (Figs 1A and 6B), which further supports it use for the same reasons. Nevertheless, experiments using more completely dephosphorylated Akt are a consideration to see if an even greater therapeutic effect size can be realized. Next, we found adding PS48 *after* Aβ peptide slightly more effective in the same *in vitro* experiments. This interesting result raises the speculation that an Aβ-induced conformational change in the Akt-PDK complex, makes the allosteric PS48 modification more effective thereafter. Preventative and treatment roles for PS48 therefore deserve further study. Finally, the AD model used here is based on cellular Aβ42 and is only one of many others. While our data are limited to this view of pathogenesis, the electrophysiology data on *ex vivo* slices exposed to soluble oligomeric Aβ species recommend that PS48 could also be tested in models emphasizing extracellular Aβ, or for that matter Tau, accumulations.

The results of published studies have been mixed with respect to the state of Akt activation in AD brain and models, reporting either over or under phosphorylation or activity. Far fewer reports specifically address the 3-phosphoinositide-dependent kinase, PDK-1 in AD or neurodegeneration. Pietri and colleagues [62, 141] found PDK-1 activity increased in neurons infected with prion protein PrPSc or in transgenic mice affected by β-amyloid pathology, as well as in AD brain. In a novel but complicated mechanism, PDK-1 overactivation is held responsible for loss of TACE-mediated APP and PrPc α-secretase cleavages, from accelerated TACE internalization. The result is an over-production of Aβ and TNF-mediated neurotoxicity and memory deficit. Accordingly, PDK-1 silencing or inhibition restored survival and memory and reversed pathology parameters. Both PrPSc and Aβ were hypothesized to stimulate PrPc to recruit Src and PI3K kinases to overactivate PDK-1. The relevance of these changes to the insulin/Akt axis was however not explored. In contradistinction, a PrPSc-like peptide (106–126) inactivated Akt and caused death in SHSY5Y and primary granule cells, outcomes confirmed in a PrPSc infected mice model. These were reversed by constitutive activation of Akt or insulin treatment [142].

It remains plausible but clinically untested if a strategy to restore Akt responsiveness to insulin, has value in prevention or treatment of AD. Based on our data targeting the PDK-Akt activation sequence, PS48 or a structurally similar allosteric analog may be a viable candidate. Importantly, PS48 does not itself over-stimulate normal insulin signaling in PCNs, nor over-activate basal Akt, lessening potential oncogenesis concerns [143, 144]. Notably, PS48 was not toxic to cells (LD50 = 250 μM). This is possibly due to the purported allosteric modulatory action of this compound, which has also been observed in various other drugs of this class [145, 146]. Other bi-aryl, halogenated carboxylic acids have a safe record in humans, for instance, Tolfenamic acid, used for the treatment of migraines [147, 148].

In support of efforts to facilitate Akt/PDK signaling, other interventions have had similar action on the insulin /Akt transduction pathwy to mitigate Aβ toxicity. For instance, α7nAcR stimulation (nicotine on PCNs) activates PI3K and pAkt to block Aβ- enhancement of mitochondrial AIF release/nuclear translocation [149], as well as block Aβ-mediated glutamate toxicity and prevent mitochondrial apoptosis [150–152]. We note with interest several recent reports that direct pharamacological activation of Akt in Aβ-injected and in 5X FAD AD mice, resolved memory impairments and synaptic LTP deficits and restored inhibited Akt to control levels [153]. Activation of Akt/PI3K in primary mouse neurons also proved protective against transfected mutant APP and improved locomotor activity in an Aβ42-drosophila model [154]. The aforementioned insulin pathway clinical trials were all supported by robust preclinical cell and animal data, e.g. GLP-1 mimetics [155–158]), IN insulin [159–162], PPAR agonists [14], as well as by epidemiological data, e.g. metformin [163, 164]. Recent reviews of these drug classes in AD prevention endorse continued clinical tials, where supported by basic studies [165–167].

Our future studies will focus on an expanded class of modified biphenyl pentanoic acids and optimization of the PS48 pharmacophore with the goal to push potency into nM range and improve cell permeability. PS48 also has a low toxicity profile in preliminary animal testing, and further clinical data will be reported separately.

## Supporting information

S1 File (PDF)

S1 Table (PDF)

S1 Raw images (PDF)
