# Correction: A PDK-1 allosteric agonist neutralizes insulin signaling derangements and beta-amyloid toxicity in neuronal cells and in vitro

**DOI:** 10.1371/journal.pone.0304731

**Published:** 2024-05-29

**Authors:** Henry Querfurth, John Marshall, Keykavous Parang, Mengia S. Rioult-Pedotti, Rakesh Tiwari, Bumsup Kwon, Steve Reisinger, Han-Kyu Lee

There is an error in affiliation 3 for authors Keykavous Parang and Rakesh Tiwari. The correct affiliation 3 is: Center for Targeted Drug Delivery, Chapman University, School of Pharmacy, Irvine, CA, United States of America.

## References

[1] Querfurth H, Marshall J, Parang K, Rioult-Pedotti MS, Tiwari R, Kwon B, et al. (2022) A PDK-1 allosteric agonist neutralizes insulin signaling derangements and beta-amyloid toxicity in neuronal cells and in vitro. PLoS ONE 17(1): e0261696. doi: 10.1371/journal.pone.0261696 35061720 PMC8782417

