# Supplementary material for: A PDK-1 allosteric agonist neutralizes insulin signaling derangements and beta-amyloid toxicity in neuronal cells and in vitro
Source: PLoS One. 2022 Jan 21;17(1):e0261696. doi: 10.1371/journal.pone.0261696 (PMC8782417; doi:10.1371/journal.pone.0261696)
Supplement: S1 File — (PDF) [file pone.0261696.s001.pdf]

Supplement figures and legends and table

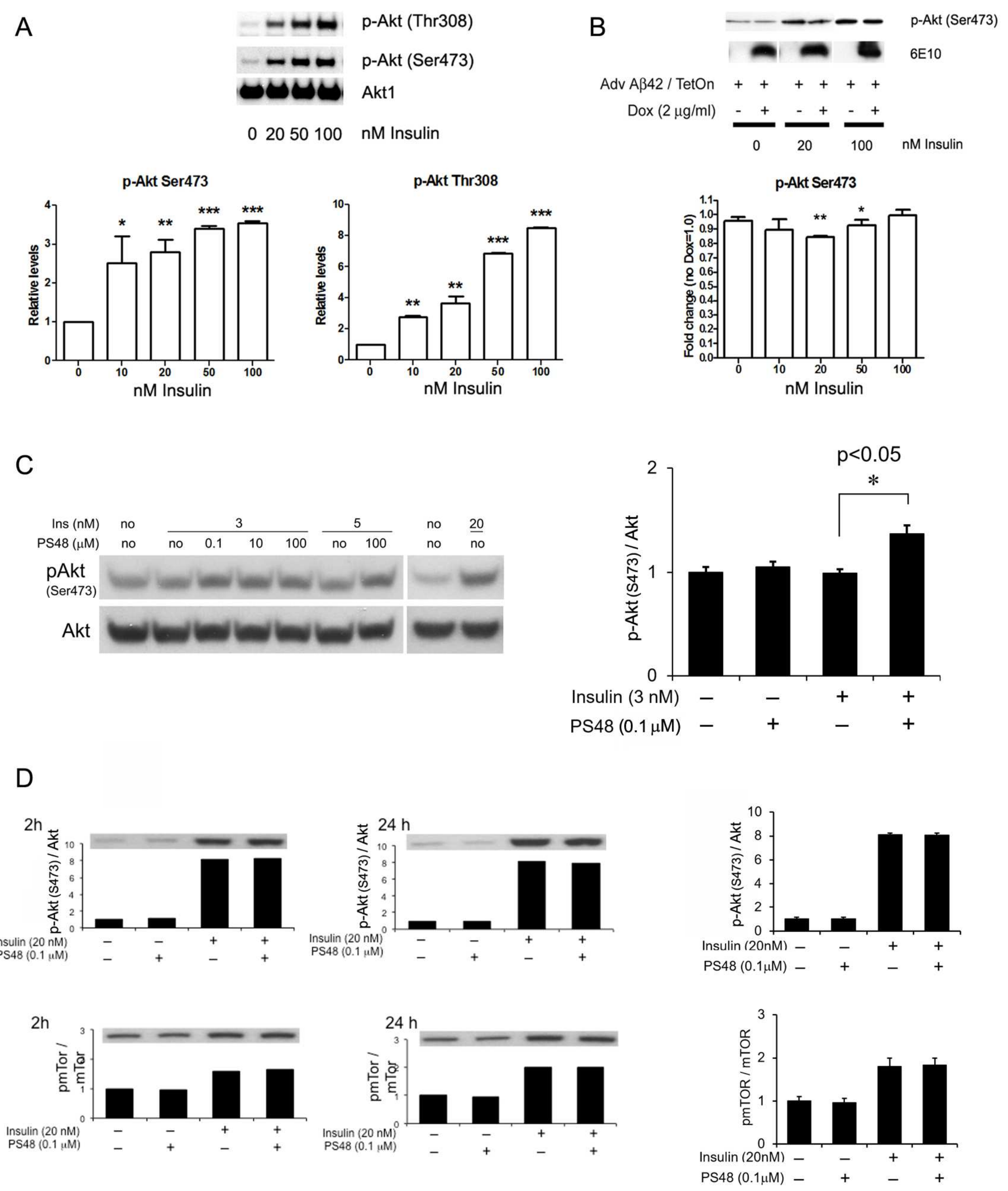

1. *Insulin resistance in PCNs and sensitizing action of PS48.* **A**. Phosphorylation of Akt is insulin dose dependent in SH-SY5Y cells. Insulin acutely is added 30 min before harvest. Near maximal responses at 'high dose' (50 to 100nM). Representative western (top) and quantification (n=3 experiments, lower) **B**. Aβ42 expression, mediated by adenovirus, inhibits insulin-stimulated Akt phosphorylation. The observed difference in phosphorylation between control (no Dox) and Aβ-expression (+ Dox) is most inhibited at 20nM insulin. However, the effect is overcome at higher doses of insulin stimulation. * p<.05, ** p<.01. **C.** At subthreshold insulin doses (3nM, 15 min, 10% serum) in N2a

cells, PS48 (0.1 to 100 μM, 2hrs) is an insulin sensitizer, boosting the phosphorylation of Ser 473 to a 20nM insulin-equivalence response. N2A cells, n=3. Quantified on right. **D**. PS48 at doses from 0.01 to 100μM for 2 hrs. or 24 hrs. does not over-activate Akt Ser473 or affect mTOR Ser2448 phosphorylations in rat primary cortical neurons stimulated with submaximal 20 nM Insulin (15 minutes prior to lysis). Western blots above and quantified below. n=2.

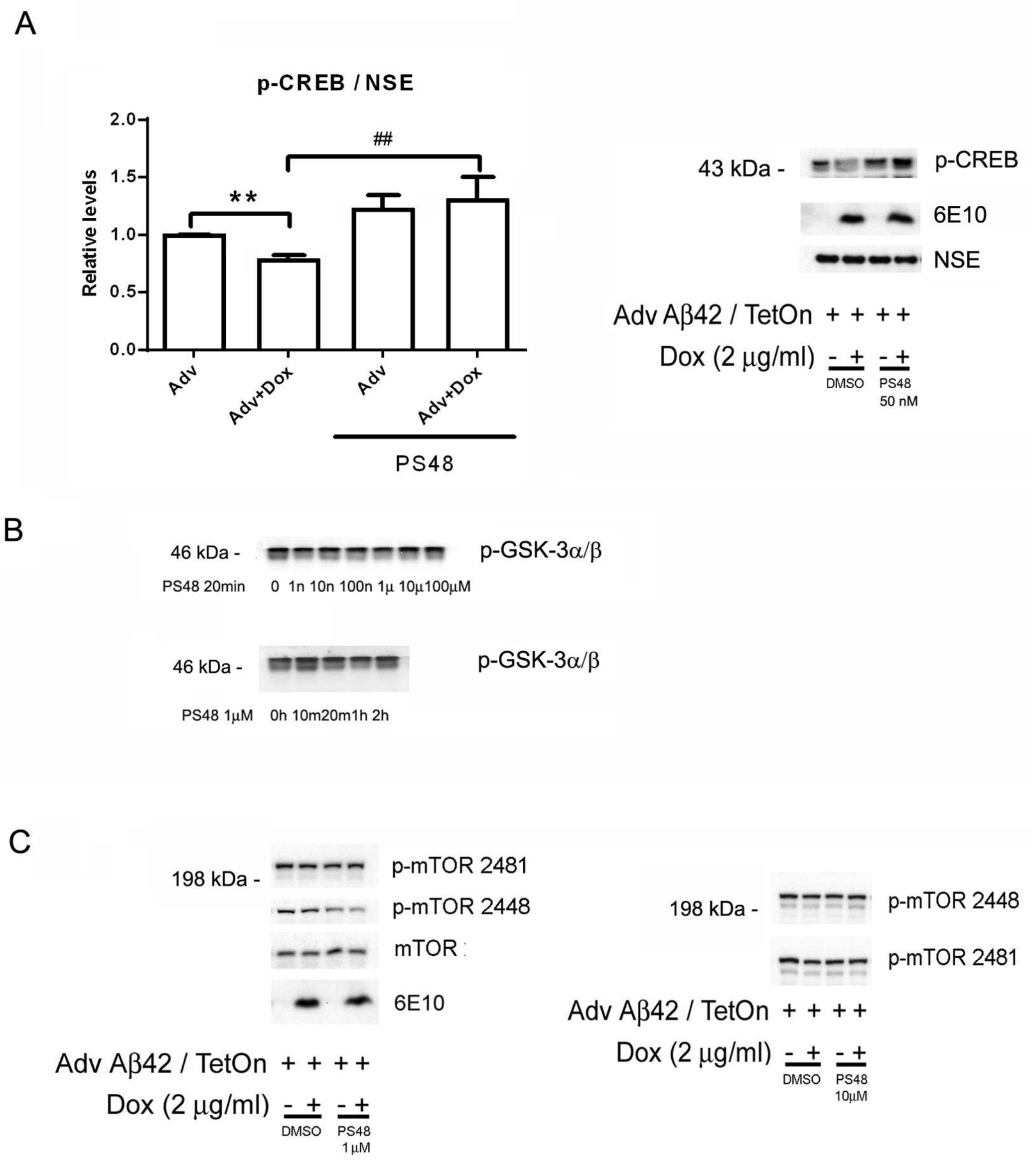

2. *Results of Aβ42-expression and PS48 on endogenous phospho-levels of Akt-effector proteins; CREB, GSK3α/β and mTOR.* **A**. Doxycycline-induced Aβ42 expression reduced p-CREB levels in SH-SY5Y cells. PS48 (50nM) mitigates the Aβ42 effect. Whole cell extracts were fractionated by Western and probed with monoclonal anti-pCREB pS133. The signals corresponding to endogenous p-CREB levels were quantified and normalized to NSE (bar graph on left). n=3 experiments. **p<.01, ## p< .01. All

cells were stimulated with Insulin 250ng/ml for 30 min before harvest. **B**. PS48 over time (1μM; 0 to 2 hours) and concentration (20 min; 0-100μM) was applied to SH-SY5Y cells. No changes in cellular phospho-GSK levels were observed. **C**. Bottom. Downstream mTOR substrate phosphorylation is unchanged by Aβ42 expression or PS48 treatment (1 or 10 μM) for 2-4 hrs. before doxycycline addition.

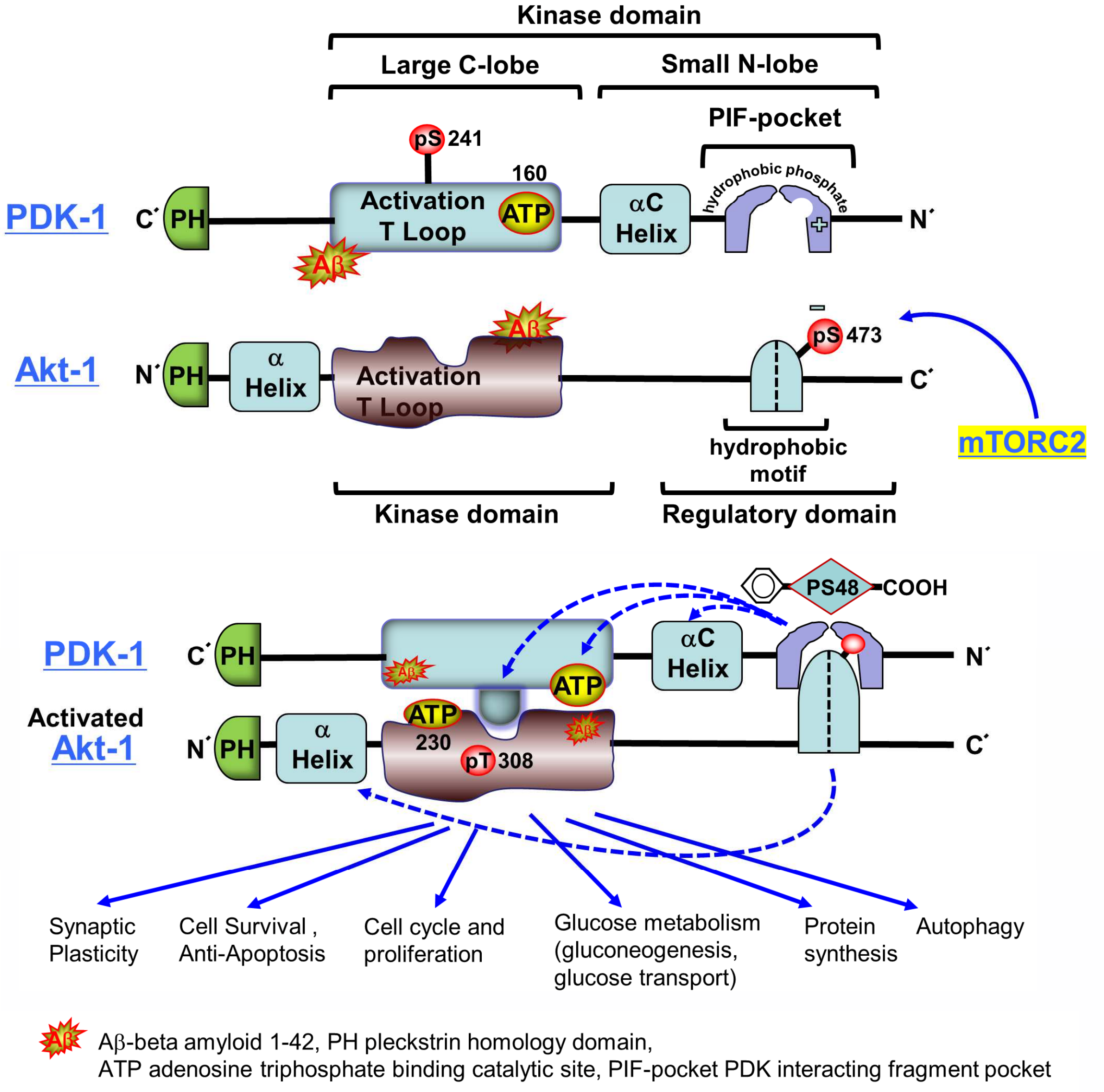

3. Hypothetical schematic of allosteric modulation of PDK-1 and activation of Akt in the presence of Aβ42

Allosteric modulators of PDK-1 such as PS48 (and PRK-2 /PIF-tide), target the PDK-interacting fragment (PIF) pocket, which is also the docking site for the AGC class kinase substrates of PDK-1 (Akt, p70S6k, SGK, PKC/PRK-2). The model shows the steric inhibitory effect of Aβ42 on insulin-stimulated Akt activation by PDK-1 (upper panel) and

allosteric modulation by PS48 to overcome it (lower panel). The model is based on the following evidence and references:

1. PS48 and analogs normalize the negative effects of Aβ42 on Akt activation in vivo and in vitro.
2. Aβ42 inhibits direct interaction of PDK-1 with Akt (pull- down data presented in previous work).
3. Aβ42 binds to both PDK-1 and Akt, in solution.
4. PS48 does not compete with Aβ binding
5. mTORC2 activity may also overcome Aβ42 provoked insulin resistance (presented in previous work).

The Aβ42 icon is for illustration only, absent molecular and physiochemical details. Moreover, it is recognized that Akt is a special case substrate of PDK-1, where docking at the PIF pocket (as shown for illustration only) is not a requirement for activation, nor is S473 phosphorylation an absolute requirement. Apparently, the binding of their PH domains to PIP3, resulting in co-localization, may be sufficient. Various intramolecular conformational changes following PIF pocket docking and/or PS48-PIF pocket binding within PDK, as well as possible domain interactions within Akt associated with activation, are indicated by dashed arrows. For detailed structural and functional properties of the activation sequence, see: [1-9]

References

1. Biondi RM. Phosphoinositide-dependent protein kinase 1, a sensor of protein conformation. Trends Biochem Sci. 2004;29(3):136-42. Epub 2004/03/09. doi: 10.1016/j.tibs.2004.01.005. PubMed PMID: 15003271.
2. Biondi RM, Komander D, Thomas CC, Lizcano JM, Deak M, Alessi DR, et al. High resolution crystal structure of the human PDK1 catalytic domain defines the regulatory phosphopeptide docking site. EMBO J. 2002;21(16):4219-28. Epub 2002/08/10. doi: 10.1093/emboj/cdf437. PubMed PMID: 12169624; PubMed Central PMCID: PMCPMC126174.
3. Engel M, Hindie V, Lopez-Garcia LA, Stroba A, Schaeffer F, Adrian I, et al. Allosteric activation of the protein kinase PDK1 with low molecular weight compounds. EMBO J. 2006;25(23):5469-80. Epub 2006/11/18. doi: 10.1038/sj.emboj.7601416. PubMed PMID: 17110931; PubMed Central PMCID: PMCPMC1679772.
4. Hindie V, Lopez-Garcia LA, Biondi RM. Use of a fluorescent ATP analog to probe the allosteric conformational change in the active site of the protein kinase PDK1. Methods Mol Biol. 2012;928:133-41. Epub 2012/09/08. doi: 10.1007/978-1-62703-008-3_10. PubMed PMID: 22956138.
5. Hindie V, Stroba A, Zhang H, Lopez-Garcia LA, Idrissova L, Zeuzem S, et al. Structure and allosteric effects of low-molecular-weight activators on the protein kinase PDK1. Nat Chem Biol. 2009;5(10):758-64. Epub 2009/09/01. doi: 10.1038/nchembio.208. PubMed PMID: 19718043.
6. Najafov A, Shpiro N, Alessi DR. Akt is efficiently activated by PIF-pocket- and PtdIns(3,4,5)P3-dependent mechanisms leading to resistance to PDK1 inhibitors.

Biochem J. 2012;448(2):285-95. Epub 2012/10/04. doi: 10.1042/BJ20121287. PubMed PMID: 23030823.
7. Stroba A, Schaeffer F, Hindie V, Lopez-Garcia L, Adrian I, Frohner W, et al. 3,5-Diphenylpent-2-enoic acids as allosteric activators of the protein kinase PDK1: structure-activity relationships and thermodynamic characterization of binding as paradigms for PIF-binding pocket-targeting compounds. J Med Chem. 2009;52(15):4683-93. Epub 2009/07/18. doi: 10.1021/jm9001499. PubMed PMID: 19606904.
8. Wilhelm A, Lopez-Garcia LA, Busschots K, Frohner W, Maurer F, Boettcher S, et al. 2-(3-Oxo-1,3-diphenylpropyl)malonic acids as potent allosteric ligands of the PIF pocket of phosphoinositide-dependent kinase-1: development and prodrug concept. J Med Chem. 2012;55(22):9817-30. Epub 2012/10/31. doi: 10.1021/jm3010477. PubMed PMID: 23106316.
9. Yang J, Cron P, Thompson V, Good VM, Hess D, Hemmings BA, et al. Molecular mechanism for the regulation of protein kinase B/Akt by hydrophobic motif phosphorylation. Mol Cell. 2002;9(6):1227-40. Epub 2002/06/28. doi: 10.1016/s1097-2765(02)00550-6. PubMed PMID: 12086620.
