# Supplementary material for: A PDK-1 allosteric agonist neutralizes insulin signaling derangements and beta-amyloid toxicity in neuronal cells and in vitro
Source: PLoS One. 2022 Jan 21;17(1):e0261696. doi: 10.1371/journal.pone.0261696 (PMC8782417; doi:10.1371/journal.pone.0261696)
Supplement: S1 Table — (PDF) [file pone.0261696.s002.pdf]

Table

**Name of Antibody**	**Manufacturer, catalog #**	**Dilution**
Akt1	Santa Cruz Biotechnology, sc-5298	1/2,000
Actin	Santa Cruz Biotechnology, sc-8432	1/10,000
phospho-Akt (Ser473)	Cell signaling, #9271	1/1000
phospho-Akt (Thr308)	Cell signaling, #13038	1/1000
Phospho-Gsk-3α/β, ser21/9	Cell signaling, #9327	1/1000
Gsk-3α/β	Cell signaling, #5676	1/1000
PDK-1	BD Biosciences, 611070	1/1000
6E10	Covance, SIG-39320	1/1000
R1282	gift from Dr. D. Selkoe	1/1000
phospho-CREB (Ser133)	Cell signaling, #9198	1/1000
mTOR	Cell signaling, #2983	1/1000
p-mTOR (Ser2448)	Cell signaling, #5536	1/1000
p-mTOR (Ser2481)	Cell signaling, #2794	1/1000
γ Enolase (NSE-P1)	Santa Cruz, sc-21738	1/1000
