# Supplementary material for: A PDK-1 allosteric agonist neutralizes insulin signaling derangements and beta-amyloid toxicity in neuronal cells and in vitro
Source: PLoS One. 2022 Jan 21;17(1):e0261696. doi: 10.1371/journal.pone.0261696 (PMC8782417; doi:10.1371/journal.pone.0261696)
Supplement: S1 Raw images — (PDF) [file pone.0261696.s003.pdf]

Fig 1A original

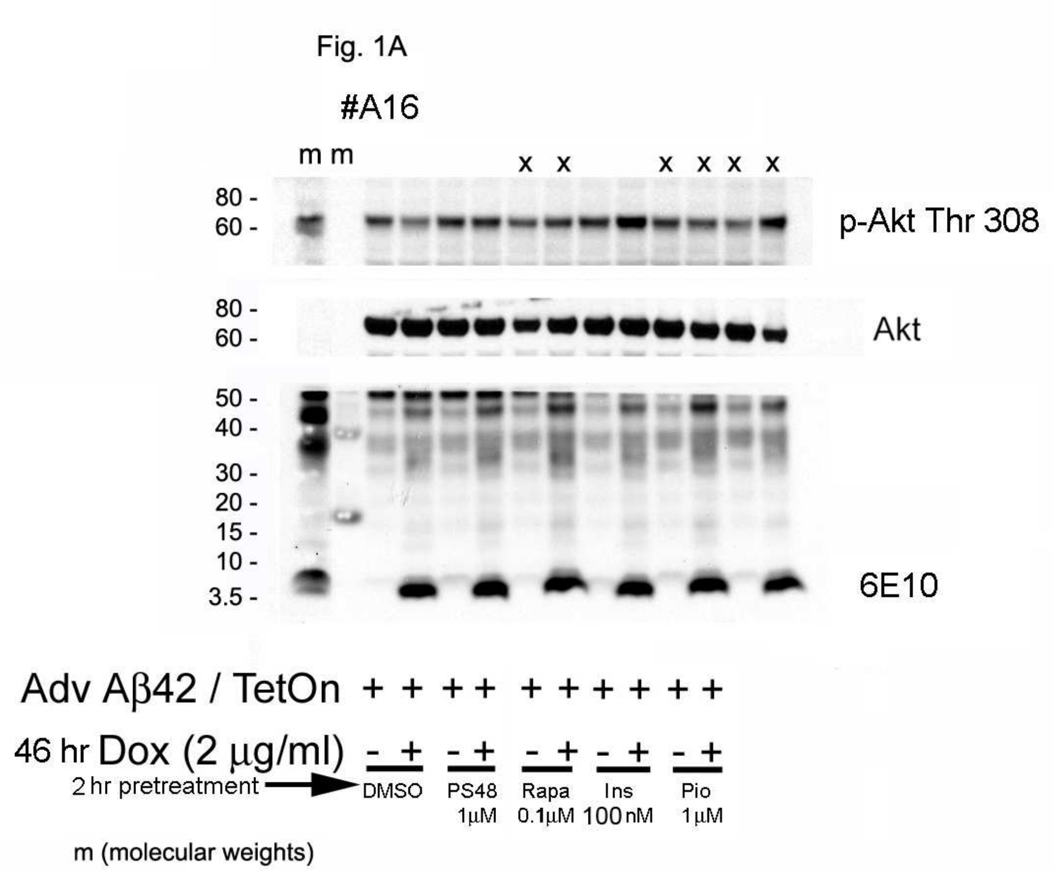

'The last lanes were left unlabeled and unpublished since they are proprietary experiments'.

Fig 1B original

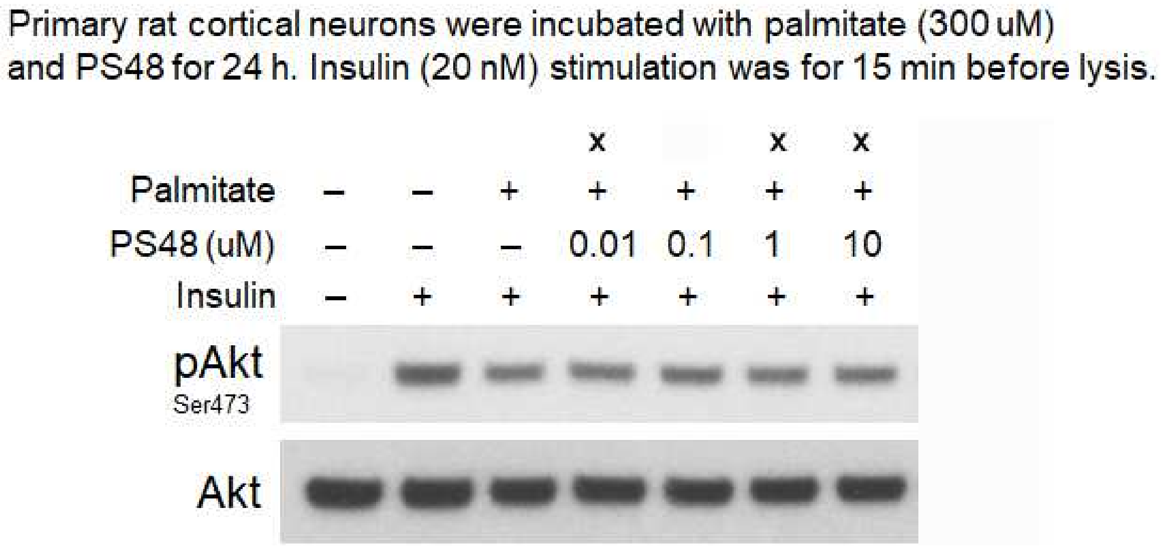

Fig 1C original

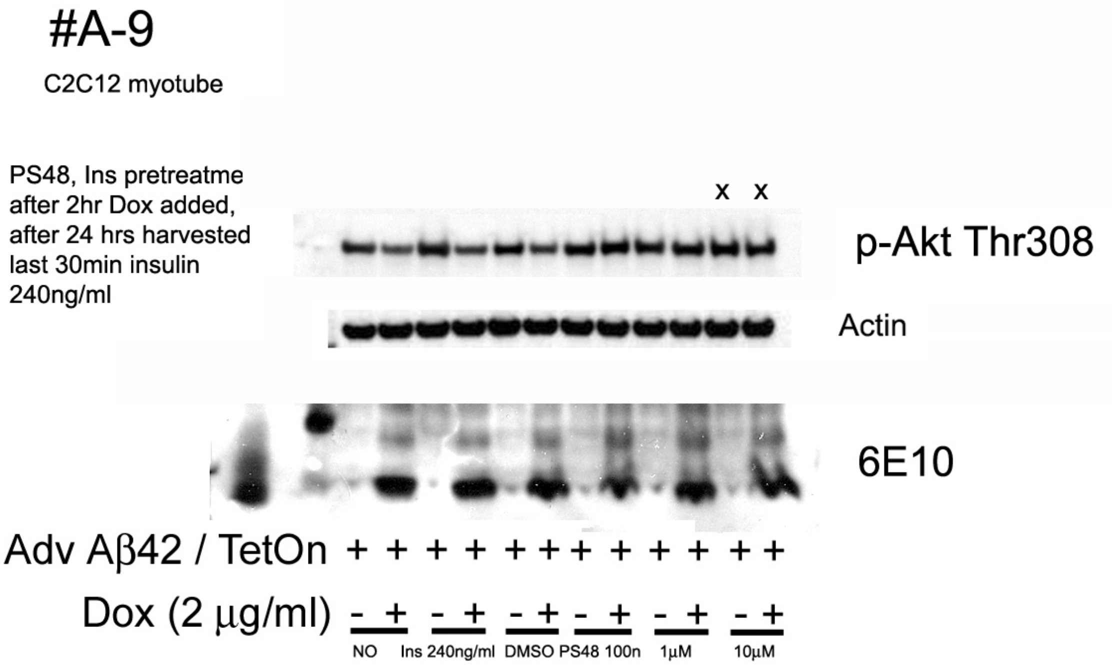

Fig 4A original

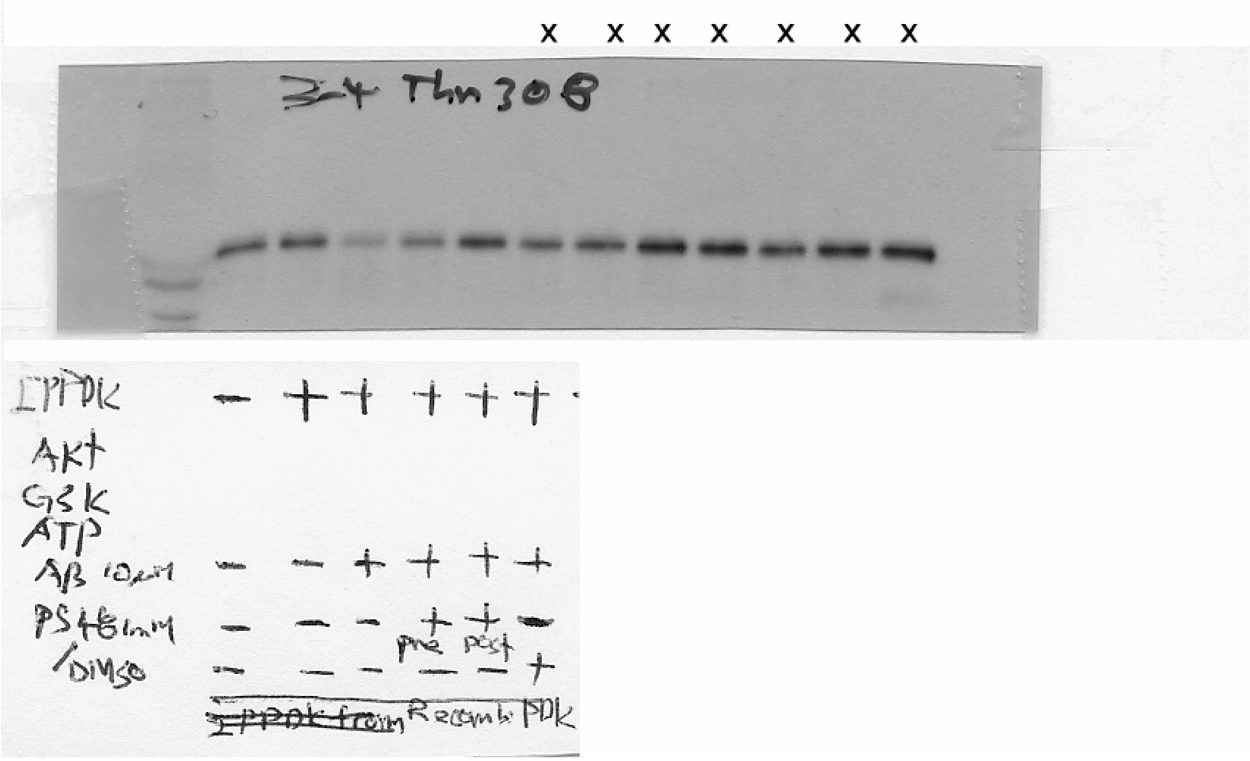

Fig 4B original

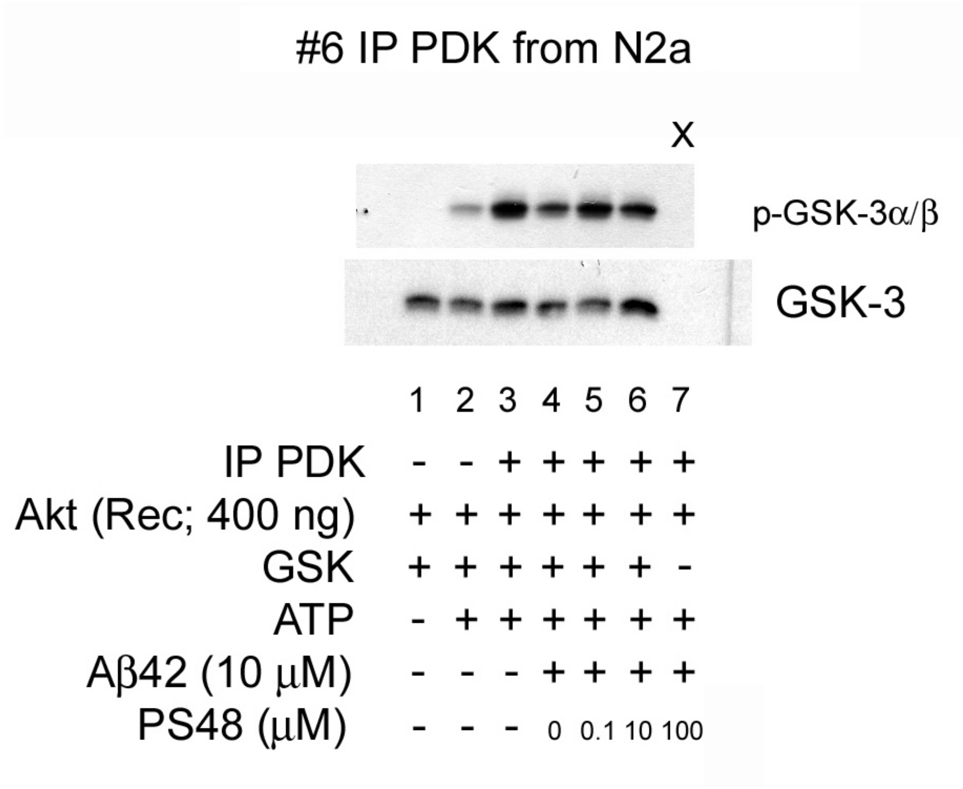

Fig 5D original

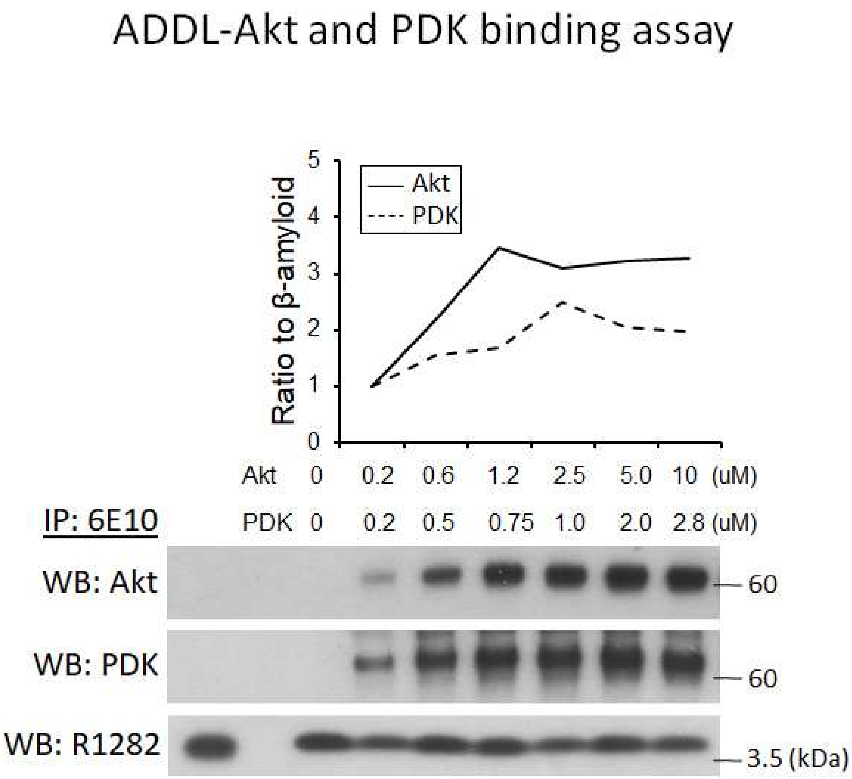

Fig 6B original

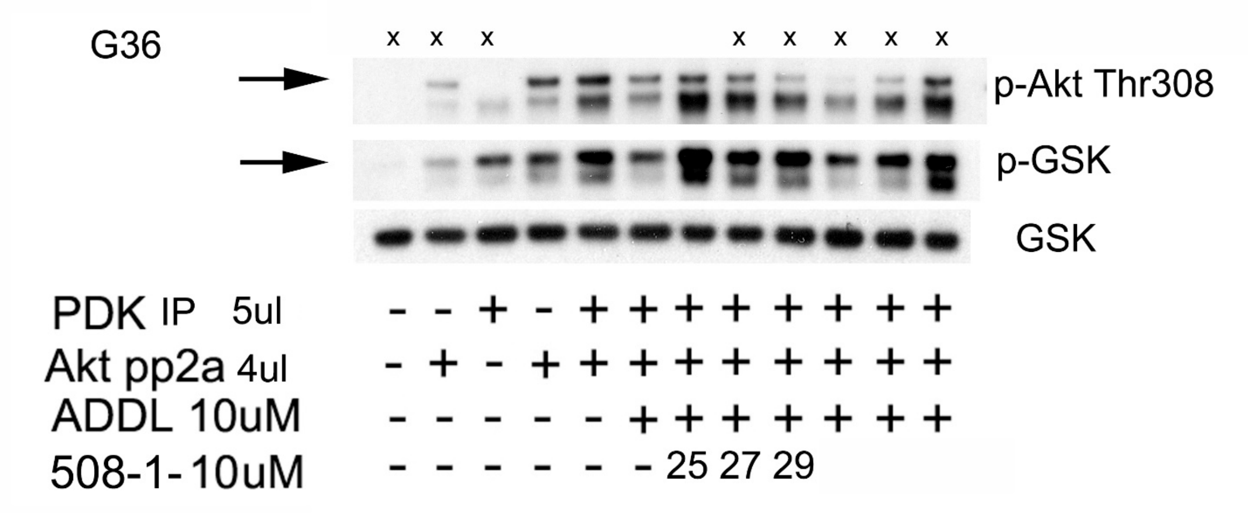

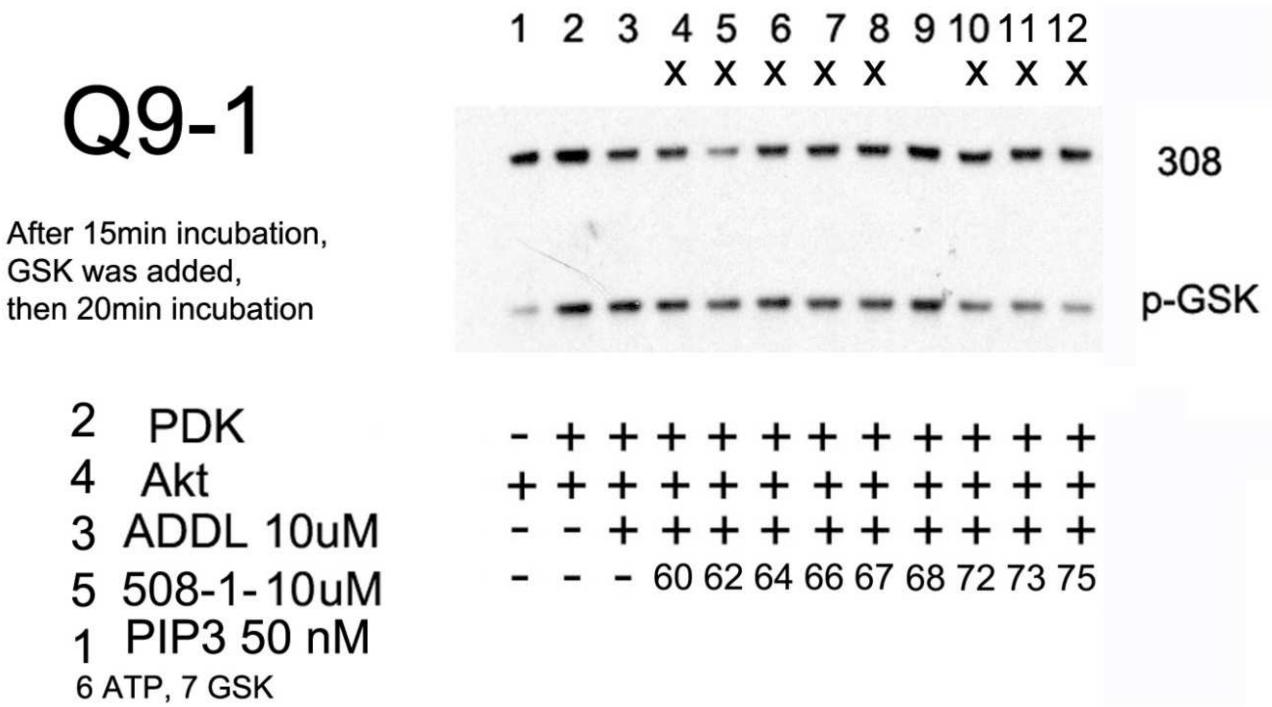

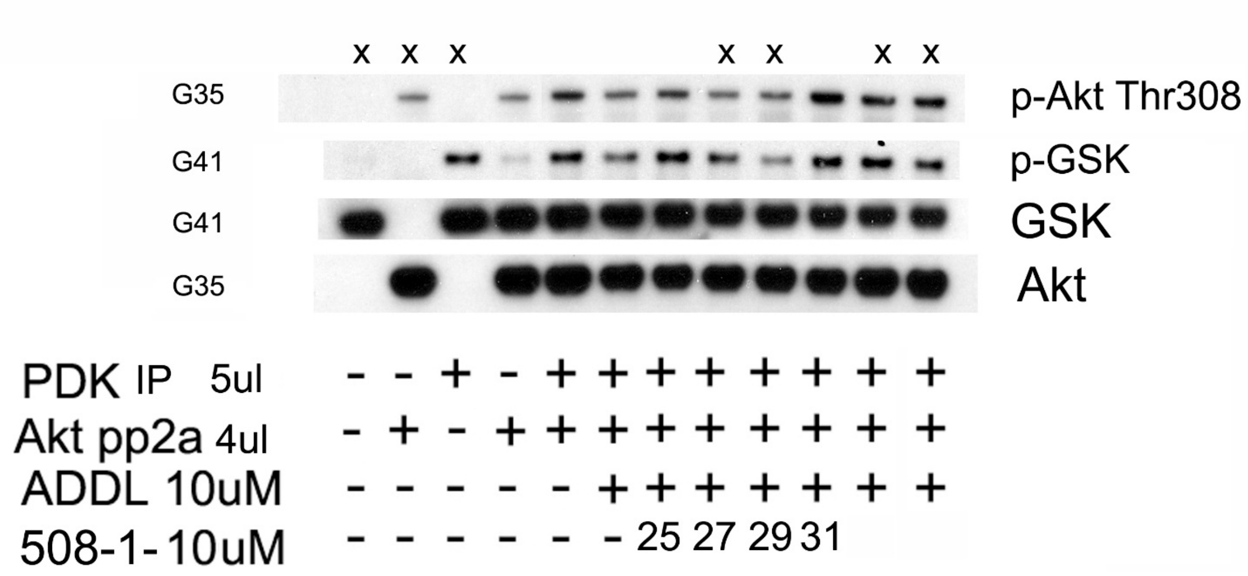

R02 Fig 6C original

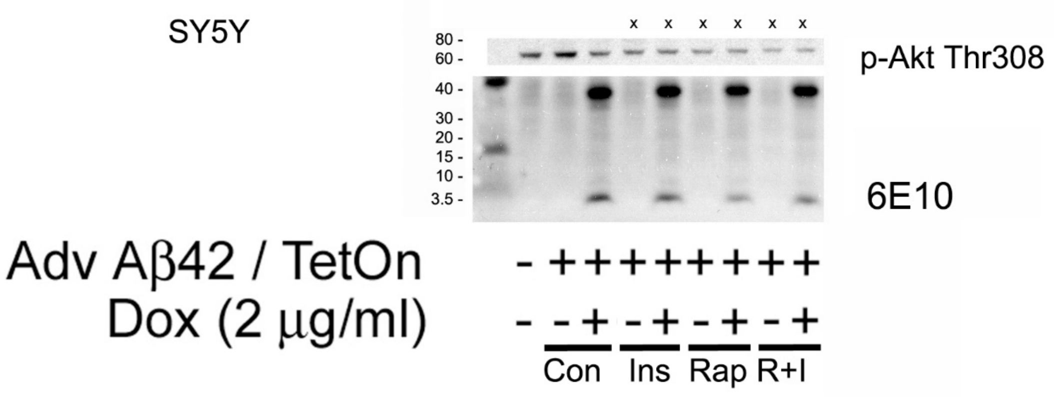

Ins: 10nM, Rapa: 500nM, 25: 1uM 20min; coincubation

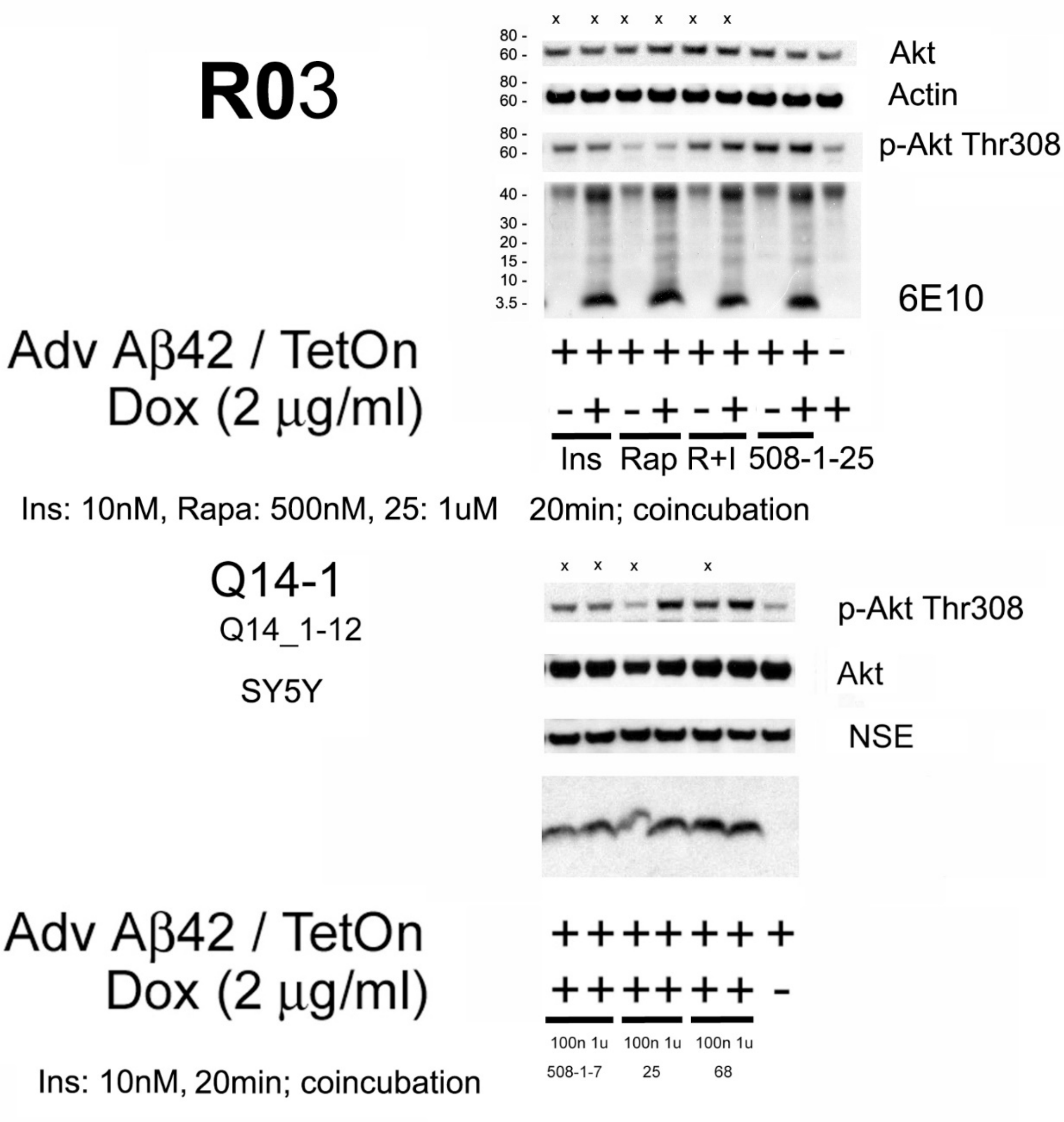

The panel in the text was taken from the 3 gels as shown, from which cmpd 25 and 68 data were extracted. These in vivo expts were run separately due to random loading of multiple drug testings and emphasis on mTOR, Rictor and rapamycin signaling changes.
